# An Overview of Artificial Olfaction Systems with a Focus on Surface Plasmon Resonance for the Analysis of Volatile Organic Compounds

**DOI:** 10.3390/bios11080244

**Published:** 2021-07-23

**Authors:** Marielle El Kazzy, Jonathan S. Weerakkody, Charlotte Hurot, Raphaël Mathey, Arnaud Buhot, Natale Scaramozzino, Yanxia Hou

**Affiliations:** 1 Grenoble Alpes University, CEA, CNRS, IRIG-SyMMES, 17 Rue des Martyrs, 38000 Grenoble, France; marielle.elkazzy@cea.fr (M.E.K.); ssweerakkody@gmail.com (J.S.W.); charlotte.hurot@gmail.com (C.H.); raphael.mathey@cea.fr (R.M.); arnaud.buhot@cea.fr (A.B.); 2 Grenoble Alpes University, CNRS, LIPhy, 38000 Grenoble, France; natale.scaramozzino@univ-grenoble-alpes.fr

**Keywords:** surface plasmon resonance, olfactory sensors, electronic noses, volatile organic compounds, odorants

## Abstract

The last three decades have witnessed an increasing demand for novel analytical tools for the analysis of gases including odorants and volatile organic compounds (VOCs) in various domains. Traditional techniques such as gas chromatography coupled with mass spectrometry, although very efficient, present several drawbacks. Such a context has incited the research and industrial communities to work on the development of alternative technologies such as artificial olfaction systems, including gas sensors, olfactory biosensors and electronic noses (eNs). A wide variety of these systems have been designed using chemiresistive, electrochemical, acoustic or optical transducers. Among optical transduction systems, surface plasmon resonance (SPR) has been extensively studied thanks to its attractive features (high sensitivity, label free, real-time measurements). In this paper, we present an overview of the advances in the development of artificial olfaction systems with a focus on their development based on propagating SPR with different coupling configurations, including prism coupler, wave guide, and grating.

## 1. Introduction

Over the last few decades, the detection of gases including odorant molecules and volatile organic compounds (VOCs) has attracted great interest and has become increasingly in demand in various field. VOCs constitute a large class of low-molecular-weight (<300 Da) carbon-containing compounds. They can exhibit odorous properties and are characterized by a high vapor pressure (≥0.01 kPa at 20 °C) and a high-to-moderate hydrophobicity [1]. These small volatile molecules have a wide range of sources, both natural (plants, animals, bacteria etc.) and anthropogenic (fossil fuels, automobile exhaust gas etc.). The majority of VOCs have inimical effects on human health such as headaches and nose, eye and throat irritation [2]. Consequently, monitoring the nature and concentration of these compounds in indoor or outdoor environments can be very important, and sometimes, vital. Additionally, they can be considered as chemical messengers. In fact, their analysis has been shown to reveal a considerable amount of information. For instance, studies in medical diagnostics have identified gases associated with different diseases such as rheumatoid arthritis, cancer, and schizophrenia [3]. Furthermore, a recent study showed the possibility of detecting viral infections such as COVID 19 through exhaled breath analysis [4]. VOC and odor analysis can also have applications in the food, beverage and fragrance industries for quality assessments. Finally, gas sensing can be very useful for security applications (detection of drugs, explosives etc.), environmental monitoring or other usages under development such as augmented/virtual reality [5]. Nowadays, the gold standard for VOC detection involves the use of trained human or canine noses or gas chromatography coupled with mass spectrometry (GC-MS). Indeed, to control the quality of raw materials or final food and perfume products, industries often have recourse to human sensory panels. Trained dogs are commonly employed for security controls or even for the detection of diseases such as prostate and breast cancers [6,7]. Although very sensitive and efficient for field studies, the use of the biological nose presents several drawbacks. For instance, human panels may yield biased subjective results and are prone to fatigue. Dogs require expensive training and their application fields are limited and sometimes risky. The second method, namely, GC-MS, is a highly sensitive and accurate analytical technique that allows separating, identifying and quantifying different VOCs in a mixture. However, analyses require skilled operators and are time consuming and expensive [8]. Therefore, there is a need for an affordable, reliable, portable and sensitive device that allows for a rapid analysis of gases including VOCs. Such a context has prompted many researchers to work on the development of alternative technology such as artificial olfaction systems that overcome the various drawbacks mentioned above.

**Figure 1:**
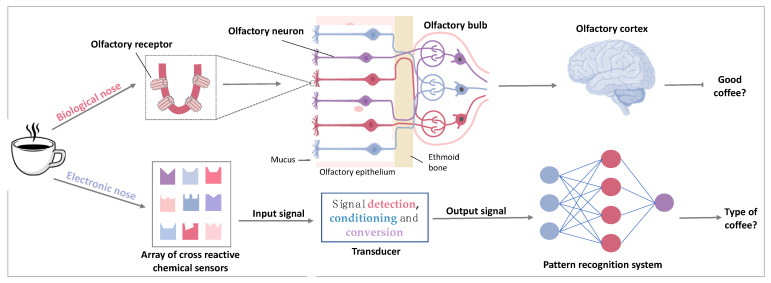
Analogy between the biological and the electronic nose (eN). Figure adapted from [11].

Herein, artificial olfaction systems include gas sensors, olfactory biosensors and an electronic nose (eN). A gas sensor or olfactory biosensor is a single-sensor device which is able to detect gases and that consists of a receptor coupled with a transducer and a data processing system. Olfactory biosensors use biomaterials as receptors. On the other hand, as stated by Julian W. Gardner and Philip N. Bartlett in 1994 [9], an eN is “an instrument, which comprises an array of electronic chemical sensors with partial specificity and an appropriate pattern-recognition system, capable of recognizing simple or complex odours”. By its very nature, the eN is, in fact, a biomimetic device that replicates the odor discrimination principle of the mammalian olfactory system. Thanks to considerable research efforts on natural olfaction, and especially, the Nobel prize winning work of Linda B. Buck and Richard Axel (1991) [10], we know that, in order to distinguish among a myriad of odors, the biological nose uses cross-reactive olfactory receptors (ORs) (about 400 different types in the human nose). This particular feature of ORs (i.e., cross reactivity or partial specificity) allows each receptor to interact with different odorant molecules with differential affinities. Therefore, in the same manner as barcodes, odors are encoded by a combination of olfactory receptors, which consequently allows the nose to have this large detection spectrum. Moreover, to transduce an olfactory stimulus, the biological odor sensor uses an extensively studied “molecular switch”: the G protein. Indeed, Buck and Axel showed that ORs belong to the large family of G protein coupled receptors (GPCRs). They are located in the plasma membrane of the cilia, i.e., the dendritic extrusions of the olfactory neurons projected into the mucus covering the olfactory epithelium. When a VOC binds to an OR, the G protein transduction cascade is initiated and the binding event is converted into an electrical signal processed by the olfactory bulb and deciphered by the olfactory cortex. Figure 1 shows the analogy between biological and electronic noses.

**Figure 2:**
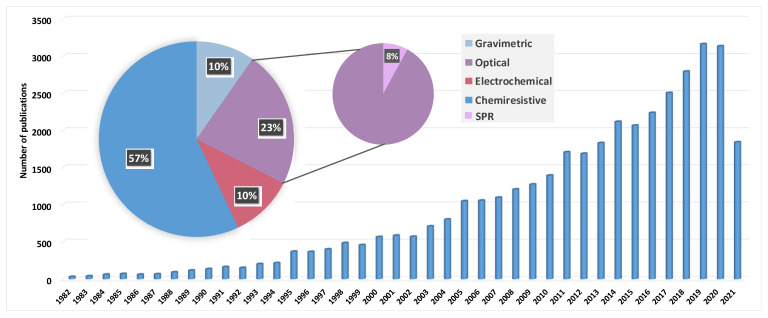
Number of publications on gas sensors and electronic noses since 1982 and the percentage of studies carried out on each type of transduction technique. The data was obtained from Scopus (Keywords used for the histogram: “gas sensor” or “electronic nose” or (gas or vapor or “volatile organic compounds”) “sensor array” or multisensor. For the pie chart: “gas sensor” or “electronic nose” or (gas or vapor or “volatile organic compounds”) “sensor array” or multisensor and (semiconduct* or chemores* or chemires* or “conducting polymer”) or (optical or “surface plasmon resonance” or colorimetric or fluorescen*) or (acoustic or piezoelectric or gravimetric) or (electrochemical).

The history of artificial odor detection starts in 1920. In their work on spray electricity and waterfall electricity, Zwaardemaker and Hogewind [12] found that the addition of odorant molecules (e.g., phenol, thymol, citrol) to water markedly raised the spray electricity which could therefore be used to detect these molecules. Subsequently, in 1950, Tanyolac and Eaton [13] attempted to detect air contaminants by measuring variations in the surface tension of a liquid drop. They showed that when contaminated air was in contact with a drop of distilled water, mineral oil or water-stabilized mercury, a considerable change in the surface tension of the drop could be observed. Based on their results, they suggested that an instrument able to classify and measure air contamination at low concentrations could be developed. The first prototype of an electronic device capable of detecting odorants was introduced by Hartman in 1954 [14]. The system was based on polarized microelectrodes as sensing elements. Following this, in 1961, using a thermistor as a transduction device, Moncrieff [15] investigated various coating materials (e.g., polyvinyl chloride, cellulose acetate, milk casein) which interacted differently with odorants. He claimed that using an array of sensors with different coatings could broaden the detection spectrum and, thus, allow for the discrimination of a large number of odors. In 1962, Seiyama et al. [16] developed a gas sensor using semiconductive thin films. The gas detection principle of their system was based on changes in electrical conductivity. A similar study was published in 1965 by Buck et al. [17]. In the same year, Dravnieks and Trotter [18] developed a vapor detector based on the thermal modulation of contact potential. Shaver [19] described a method to enhance the sensitivity of a tungsten oxide gas detector by the addition of a catalytic material such as platinum in 1967. The following year, Taguchi fabricated the first metal oxide semiconductor (MOS) gas sensors for home and industrial usage employing tin oxide as sensitive coating material, which he subsequently patented in 1971 [20]. His company, Figaro Engineering Inc., became the main manufacturer of MOS gas sensors. In 1979, Wohltjen and Dessy [21] introduced the first surface acoustic based gas sensor. However, it was not until 1982, with Persaud and Dodd [22], and then in 1985, with Ikegami and Kaneyasu [23], that the first electronic nose systems based on an array of intelligent chemical sensors emerged. In order to understand the discrimination mechanism of the sense of smell, Persaud and Dodd designed a model of the nose using three Figaro sensors with a differential response spectrum. As a result, their device was able to distinguish among a wide variety of odors, and highlighted the importance of nonspecific interactions in the odor discrimination mechanism. As shown in Figure 2, over the following decades, an exponential number of studies were carried out in order to develop gas sensors and electronic noses. Different sensor systems employing chemiresistive, electrochemical, piezoelectric, and optical transducers [24] have been deployed and assembled in an array to construct eN systems.

To date, most eN systems have used chemical layers (metal oxide semiconductor, polymers, etc.) as sensing elements. However, these systems suffer from limited diversity of sensor coatings and poor selectivity. To improve the odor sensing performance, the latest trend consists of using natural biological elements such as ORs and odorant binding proteins (OBPs) or their analogues, such as peptides as sensitive materials [25,26]. Indeed, the sensitivity and selectivity of such receptors have been naturally improved and optimized by millions of years of evolution, making them ideal candidates for odor detection. However, integrating them into an electronic device and maintaining their bioactivity in nonoptimal conditions is very challenging. Promisingly, great improvements have been made in this novel field of olfactory biosensors and electronic noses [25,26,27,28,29,30].

A large number of reviews have presented the operating principles of the various sensor systems that have been developed so far for VOC and gas detection [8,24,31,32,33,34,35,36]. In addition, several reviews have focused on the development of gas sensors and eNs based on the main techniques, namely, chemiresistive [11,37,38,39,40,41], gravimetric [42,43], amperometric [44], optical fibers [45], colorimetric and fluorometric [46]. However, to the best of our knowledge, no review has emphasized the development of gas sensors, olfactory biosensors and eNs based on another popular technique, namely, surface plasmon resonance (SPR). Indeed, SPR offers many advantages compared to other techniques, including label free measurement with quantitative and qualitative data, real-time monitoring with information on the affinity and the kinetics of the studied interaction, compatibility with multiplex and high-throughput analyses, reusable sensor chips, and repeatable measurements. Accordingly, in this review, we aim to first give a brief overview of artificial olfaction systems based on various sensor systems, and then a focus on the advances made using SPR.

After this introduction, the second section will review the most common sensing systems currently employed for VOC and gas detection. The third part will be dedicated to advances in SPR-based gas sensors, olfactory biosensors and eNs. It includes a brief description of the theoretical principles of the SPR technique followed by an overview of research works using SPR with different coupling configurations.

## 2. Gas Sensors and Electronic Noses Based on Various Sensing Systems

As stated, many ingenious systems with different types of sensing materials and transduction techniques have been devised and studied. In the following section, we present a brief overview of the most commonly used sensing platforms for VOC and gas detection. For each system, we will underline the transduction principle, strengths, weaknesses, and present some illustrative examples from the literature.

### 2.1. Chemiresistive Sensors

This category mainly includes three types of gas sensors, i.e., using MOS, conducting organic polymers (CP) and carbon-based materials [47]. These sensors have a common operating principle whereby the binding of VOCs induces a variation in the electroconductivity. They also have a similar structure that essentially consists of an active layer deposited on a substrate with two electrodes to measure changes in resistance upon exposure to target molecules [39,40,48]. In the following part, popular MOS sensors and CP-based sensors will be discussed more in detail. Gas sensors using carbon material (graphene, carbon-nanotubes, etc.) are not discussed here. More information can be found in recent reviews [49,50].

#### 2.1.1. MOS Sensors

MOS-based sensors are the most commonly used systems for gas and VOC detection among all the sensing technologies [39]. They were first manufactured and marketed by Taguchi in 1968 for gas leak detection [31,35]. These sensors are typically made of a ceramic substrate coated with either n-type (mainly SnO_2_, TiO_2_, ZnO) or p-type (e.g., NiO) metal-oxide semiconducting film between two electrodes. The ceramic substrate usually contains a heating element that allows the device to reach its operating temperature, generally ranging between 200 and 500 °C [32]. The transduction mechanism of these sensors is based on variations in their conductivity or resistance upon gas molecule binding, which was well addressed in a recently published review [51]. Various factors, such as the bulk resistance, surface effect, grain boundary and contact between the grain interface and the electrode, can affect the electrical properties of gas sensing materials in MOS-based sensors. The detection spectrum and sensitivity of the sensors can be tuned by doping the semiconductor film with noble catalytic metal (e.g., Pt, Pd) [52] or by modifying the working temperature. The grain size, the thickness, and the microstructure and morphology of the coating film can also affect the binding affinity of the device [32,40,53].

These sensors are attractive candidates for eN as they offer high sensitivity with fast response and recovery times. They are also robust and easy to use. Moreover, advances in micro- and nano- fabrication technologies have enabled low-cost production of miniaturized sensor arrays [41,54]. The major drawbacks of these sensors are the lack of selectivity, their susceptibility to humidity and the high operating temperature which leads to high power consumption and reduced lifespan [39,55]. Nevertheless, great efforts have been made to overcome these drawbacks. Low-power microheaters have been designed and new porous structures have been explored [39,54,55]. Moreover, room temperature operating MOS sensors have been developed following different strategies, and involve the use of metal oxide nanostructures such as nanowires, nanotubes and nanobelts [56,57]. MOS sensors and particularly SnO_2_-based systems have been extensively studied, miniaturized and combined into arrays for the detection of a large panel of VOCs. Hundreds of outstanding works on experimental and commercial eN systems can be cited. However, this not being the subject of the present review, more details about these systems can be found in the cited reviews [11,34,39,41,54,55,58].

#### 2.1.2. Conducting Organic Polymers Sensors

CP based sensors have received considerable attention since the early 1980s [59]. They are probably the most widely used systems for VOC detection after MOS sensors, and were used in the earlier generations of electronic nose systems [35,36]. CP based sensors are generally composed of a substrate (e.g., glass microscope slide, silicon wafer), on which a film of conducting polymer is deposited between two parallel or interdigitated electrodes [31]. Intrinsic conducting polymers (ICPs) such as polypyrrole, polyaniline, polythiophene and their derivatives have been typically employed for sensor applications [37]. They are usually deposited by electro-polymerization [35]. As for MOS sensors, the transduction principle of these devices relies on variations in the conductivity of the sensors in the presence of VOCs. Several studies have investigated the interaction between the ICPs and the target molecules and suggested different mechanisms [37,38,60]. Reversible modulation of conductance is detected by measuring variations in the current flowing through the polymer when a voltage is applied across the electrodes [31]. The sensing performance of the CPs can be adjusted by modifying the polymer molecular structures, changing the dopants and incorporating a second component into conducting polymers [37]. The addition of a second component gives rise to an original new category of sensing elements called hybrid or composite conducting polymers (CCPs). Further information and examples of CCP-based sensors can be found in the following reviews [37,61].

Unlike MOS sensors, CP-based systems can operate at room temperature, and thus, consume less power. They also exhibit good sensitivity and have short response times [37]. In addition, they are easy to fabricate and resistant to poisoning [8,24]. However, these devices suffer from a lack of selectivity and baseline drift. Moreover, their sensitivity can be affected by humidity and temperature and they can be overloaded by some VOCs resulting in a short lifetime [24,35,54]. Hundreds of papers about CP-based sensors and eNs can be found in the literature [37,38,54]. CP-based gas sensor arrays have been developed for many applications. For instance, Yu et al. have designed a portable array of polypyrrole sensors for the analysis of diabetic patient’s breath [62]. Li et al. detected aromatic organic compounds using nanofibers of conducting polyaniline [63]. CP have also been used as sensitive coatings and combined with different sensing platforms such as quartz crystal microbalance [64] and field effect transistors [65].

### 2.2. Electrochemical Sensors

This family of sensors includes three main categories classified according to their measurement approaches: amperometric, potentiometric and conductimetric/impedimetric sensors [44]. These electroanalytical techniques generally involve monitoring the modulation of an electrical property (current, potential, conductivity or impedance) associated with the interaction of odorant molecules with the working electrode [24]. The working electrode is usually made of gold or platinum and covered with sensing materials, for example, in certain cases, a porous membrane that acts as a transport barrier [35].

These sensors have the advantages of being robust and can function at room temperature [24]. They are also low cost, have low power consumption and can be miniaturized [66], which are all suitable characteristics for eN systems. Additionally, the reactivity of these gas sensors can be customized by adding metal layers, polymers or biological sensing materials to the working electrode surface [34]. However, due to their sensing methodology, some of these sensors have a narrow detection spectrum with a high sensitivity only to a limited number of electrochemically active gases [36]. Several groups have explored the potential of different categories of electrochemical sensors for the detection of VOCs and odorant molecules. For instance, Buttner et al. [67] have demonstrated the usability of an amperometric sensor for in situ detection of explosives in soil. Barou et al. [68] presented a proof of concept for the detection of odorant molecules using square wave voltammetry. Liu et al. [69] designed an olfactory biosensor based on electrochemical impedance spectroscopy (EIS). Also using EIS technique, Hou et al. [70] were able to detect odorant molecules by monitoring the electrical properties of a Langmuir-Blodgett film containing OBPs. In another study [71], employing the same electroanalytical method, the team reported a novel odorant detection strategy using a rat olfactory receptor. As a part of the European project SPOT-NOSED, Akimov et al. [72] worked on the development of nanobiosensors that consist of a single olfactory receptor anchored between nanoelectrodes that detects odorant binding using EIS.

### 2.3. Field Effect Transistor (FET)

There are several types of FET gas sensors, including thin-film transistor, catalytic metal gate FET, suspended gate FET, capacitively coupled FET and horizontal floating-gate FET. The transduction principle of these devices is mainly based on the modulation of the threshold voltage or the drain source current. Each type of FET sensor has a specific structure, sensing mechanism and characteristics with different advantages and drawbacks. Hong et al. [73] recently published a paper that explains and reviews the operating principle, features and performance of each type of FET sensor.

Many research groups have studied and explored this type of sensor for VOC detection applied to different areas and using various types of sensing materials. For example, Haick’s group has extensively worked on the development of silicon nanowire field effect transistors (SiNW FET). The SiNW FET surfaces were modified with different types of organic molecules in order to detect different kinds of VOCs and specially disease biomarkers [74,75,76]. Park’s team developed a highly sensitive FET based bioelectronic noses using single walled carbon nanotubes or polypyrole nanotubes conjugated with human ORs [65,77]. Johnson’s group designed and studied VOC sensor arrays using DNA-decorated carbon nanotubes FETs [78,79,80] and graphene FETs [81]. Kotlowski et al. [82] described an olfactory biosensor employing reduced graphene oxide FET functionalized with OBPs. Liao et al. [83] demonstrated that organic thin-film-transistors are suitable for electronic nose development.

### 2.4. Gravimetric or Piezoelectric Sensors

Two types of piezoelectric sensors are mainly used for VOC and gas detection: surface acoustic wave (SAW) sensors [8,35] and bulk acoustic wave (BAW) also called quartz crystal microbalance (QCM). A SAW sensor, in delay line configuration, basically consists of two inter-digitated transducers (IDTs) placed on top of a piezoelectric substrate such as quartz or Lithium niobate. To detect target molecules, a sensitive membrane (e.g., conducting polymers, lipids, biomolecules, etc.) is deposited between the IDTs [8]. A QCM sensor comprises a quartz disc coated with two gold electrodes connected to either side of the disc and a layer of sensitive material [35]. Despite their structural differences, both sensors have similar transduction principles. They detect odorant molecules by measuring variations in the resonant frequency caused by a change in mass after VOCs adsorption [8,31,32]. When an alternating voltage is applied across the piezoelectric element, it oscillates at a specific frequency driven by its mechanical properties [31]. This produces 2-dimentional acoustic waves (Rayleigh waves) that propagate along the surface at a frequency between 100 and 400 MHz in SAW sensors. Whereas, in QCM devices, 3-dimentional waves that travel through the bulk at a frequency of 10 to 30 MHz are generated [31].

QCM and SAW sensors have short response time and they are able to work at room temperature. Moreover, the detection spectrum of these devices can be tailored by modifying their sensitive membrane (the sensing materials) [8]. However, they suffer from complex circuitry and limited multiplexing capacity for large sensor array system. Additionally, the coating technologies are poorly controlled resulting in sensors having poor batch-to-batch reproducibility [31]. To tackle this issue, Chevalier et al. [84] showed that diamond nanoparticles can promote homogenous and reproducible coating of SAW sensors. A large number of studies have focused on the development of SAW and QCM based gas sensors and eNs using various sensitive materials. Rapp et al. [85] presented an improved array of eight SAW sensors for the detection of organic gas and an in-built multiplexing technique that allows an easy optimization of signal to noise ratio. They expanded the choice of coatings for the SAW sensors and improved the sensor to sensor reproducibility for a certain coating material. Matatagui et al. [86] recently designed a portable low-cost eN based on SAW sensors and using ferrite nanoparticles as sensing materials for the detection of BTX (benzene, toluene and xylene), which are hazardous gases. Panigrahi et al. [87] worked on the detection of a VOC associated with *Salmonella* contamination in meat using a QCM system coated with synthetic polypeptides. Compagnone et al. reported a QCM sensor array using peptide modified gold nanoparticles for the detection of food aromas [88]. In another study [89], they have investigated the use of metallo porphyrins coated QCM platform for quality control of chocolate. Likewise, Di Natale et al. [90] designed an array of eight QCM sensors coated with metallo porphyrins for the detection of lung cancer. Park’s group [91] and Wang’s group [92,93] have developed QCM and SAW olfactory biosensors by employing ORs as sensing materials. Furthermore, several studies have explored the performance of QCM based sensors coupled to molecularly imprinted polymers (MIPs) for the detection of VOCs [30].

Other types of gravimetric sensor systems based on film bulk acoustic resonator [94,95,96], cantilevers [97,98,99,100,101], capacitive micro-machined ultrasonic transducer [102,103] have also been explored and optimized for the detection of VOCs. The following reviews [34,42,43,104] provide more details about these sensors and bring together different research articles that focus on the development of this technique.

### 2.5. Optical Sensors

This category of sensors detects odorants by measuring variations in the optical properties (e.g., refractive index, fluorescence, absorbance) of the sensing material by monitoring light properties modulation (e.g., wavelength, intensity, phase). They involve the use of a large assortment of techniques including different categories of optical fibers and a diversity of light sources and light-sensitive photodetectors [24]. Depending on the operating principle (i.e., the optical property that is monitored), it is possible to distinguish among several types of optical sensors, each having advantages and drawbacks. It is important to mention that optical spectroscopy (near infrared, infrared, Raman, etc.) is also very promising for gas sensing. Herein, it is not in the scope of this paper and thus will not be considered. More information can be found in a recently published review [105].

The simplest optical sensors effective for electronic nose development are colorimetric sensors. These sensors are based on the measurement of UV−vis absorbance or reflectance and involve the use of chemoresponsive dyes (chromophore) such as metalloporphyrins that will change color upon exposure to VOCs [106]. They have the advantages of being low cost, easy to manufacture and allow real-time multiplexed monitoring of VOCs. However, their main drawback is that they do not offer quantitative measurements [107]. Suslick’s group pioneered this technique. They have extensively developed this type of sensors with a large number of published articles where they showed efficient detection of VOCs with very low detection limit for different applications [46]. Hou’s group also developed a colorimetric sensor array for the detection of aldehydes and lung cancer biomarkers [108] and for the discrimination of Chinese liquors [109].

Fluorometric or fluorescent sensors are more sensitive than colorimetric sensors and involve the use of fluorophores. They can be categorized into different types based on the fluorescence parameter that is measured (e.g., fluorescence intensity, anisotropy, lifetime, emission and excitation spectra, fluorescence decay, and quantum yield) [24,46,110]. Walt and co-workers pioneered multiplexed fluorescent sensors combined with optical fibers [111]. Indeed, fiber optic platforms are widely used for optical sensor development thanks to their attractive features, including remote and multiplexed sensing capability, biocompatibility, miniaturized structure, light weight, flexibility and immunity to electromagnetic interference [112]. Another main advantage of these systems lies in the temporal response obtained with the kinetic information compared to the equilibrium response obtained with most other sensing technologies. In the field of VOC detection, Walt et al. [111,113] developed an array of optical fibers with a solvatochromic dye (Nile red) encompassed in different polymer matrices with diverse polarity, flexibility, hydrophobicity, pore size and swelling tendency in order to obtain sensors that interact differently with VOCs. The sensitive polymer/dye combinations were deposited at the distal end of the fiber. Changes in the fluorescence intensity at a given wavelength upon the exposure to VOCs were recorded over time thanks to a CCD camera. In another study [114,115], they developed an array of fluorometric fiber optic-based sensors (FOS) where the fluorescent dyes were incorporated into different classes of microbeads. The beads were then immobilized in microwells at the tip of the imaging fiber. Kang’s group also developed fiber optic-based fluorometric sensors for VOCs detection [107,116,117]. In particular, the team presented an array of five FOSs using four different types of solvatochromic dyes and two different polymers to form sensitive membranes. The sensing materials were deposited on side-polished optical fibers and pulse width modulations were measured as a response to VOCs [116]. More details and examples about colorimetric and fluorometric sensors can be found in the following reviews [35,46,114,118].

Another important family of optical sensors is based on surface plasmon resonance and involves the excitation of surface plasmons that are extremely sensitive to variations in the refractive index of the sensing materials. In 1982, Nylander et al. [119] investigated the possibility of employing SPR as a transduction technique for gas detection. Using an organic film as a sensing material, their system demonstrated a sensitivity to halothane in the parts per million (ppm) range. Since then, this optical sensing technique has gained substantial popularity. Owing to its prominent attractive features, namely, high sensitivity, label free detection and real time measurements, SPR constitutes a very powerful tool for sensor development comparing to other optical techniques. It has proven to be very useful for monitoring and studying interactions and affinities especially between biological elements (e.g., antibody-antigen, ligand-receptors). Consequently, SPR has been extensively employed for a large number of applications including diseases diagnosis, drug discovery and other bioanalysis [120,121]. Additionally, SPR sensors have been used for the detection of chemical species such as VOCs. Indeed, many research groups have developed efficient gas sensors, olfactory biosensors, and electronic nose systems using SPR as sensing technique. This will be the focus of the following section of the review. To illustrate the progress in this domain, examples of studies with different SPR coupling configurations will be presented and discussed.

## 3. Propagating SPR-Based Gas Sensors and Electronic Noses

The SPR phenomenon was first observed by Wood [122] in 1902. In his study, he pointed out inexplicable peculiarities in the spectrum of light diffracted by a diffraction grating. To understand this phenomenon, in 1941, Fano [123] re-examined Wood’s observations and showed that the anomalous diffraction pattern was caused by the excitation of “polarized quasi-stationary waves” present at the surface of the metallic gratings. In 1952, Pines and Bohm [124] suggested that the energy losses of fast electron passing through foils were caused by the excitation of plasma oscillations or “plasmons” i.e., oscillations of the electronic density in the conducting media. Hereafter, this energy loss and its association with surface plasma oscillations were studied by Ferrell and Stern [125,126], Ritchie [127], Powell [128] and many others. In 1968, Otto [129] presented a method for the excitation of nonradiative surface plasma waves and showed that it resulted in a strong attenuation of the reflected light intensity. Moreover, in the same year, Kretschmann and Raether [130] described another configuration that enabled the excitation of the nonradiative surface plasmons (SPs).

A plasmon corresponds to the collective oscillation of the free electrons in a noble metal [131]. Surface plasmons are collective oscillations of electrons that take place at the interface between two media having dielectric constants of opposite signs typically a metal (e.g., gold, silver) and a dielectric (e.g., air, water) [132]. The SPs are not arbitrary events, they occur upon the excitation or coupling to an electromagnetic photon wave (i.e., light). In fact, when a photon beam interacts with the free electrons of a metal, these electrons will respond by coherently oscillating in resonance with the light wave. This phenomenon is known as surface plasmon resonance and corresponds to the excitation of the SPs.

SPs can be classified into two categories: propagating or localized.

**Figure 3:**
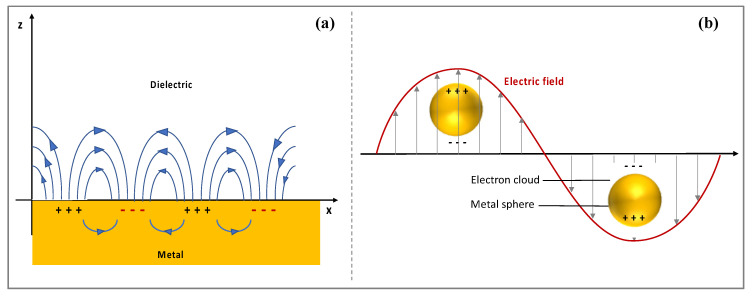
Schematic representation of (**a**) propagating surface plasmon (SP) and (**b**) localized surface plasmon.

Propagating SPs, also known as surface plasmon polaritons (SPPs) or surface plasma waves (SPWs), are typically produced at the surface of thin metallic layers. SPPs can be considered as electromagnetic waves that propagate along the planar surface of a metal interfacing a dielectric (Figure 3a). The excitation of the SPs in such structures requires the use of coupling elements (e.g., prism, waveguide, gratings) that allow to achieve resonance or matching conditions leading to SPR.

On the other hand, localized surface plasmon resonance (LSPR) occurs when light interacts with metallic nanostructures (e.g., gold nanoparticles) that are smaller than the incident wavelength [133,134]. The electric field of the light causes the localized free-electrons in the nanostructure to oscillate with a specific frequency. When the electron cloud is displaced relative to the nuclei a restoring force, generated by the Coulomb attraction between the electrons and the nuclei causes the electron cloud to oscillate relative to the nuclear framework [135] (Figure 3b). This has three main consequences: an enhancement in the local electromagnetic field near the particle’s surface and a strong light scattering as well as a sharp spectral absorption with a maximum at the plasmon resonant frequency [136]. For gold nanoparticles (size ranging from few to hundreds of nanometers), a strong absorption pic is observed in the visible light leading to their red color in solution [136]. Unlike the SPR phenomenon, which takes place at the surface of a metallic film, LSPR does not require coupling elements and does not propagate hence its localized character. However, it is likewise sensitive to changes in the local dielectric environment. In particular, the extinction peak (namely the resonance wavelength) is highly affected by the refractive index of the surrounding. Thus, for sensing applications, molecular interactions occurring at the surface of the nanoparticles are typically detected by monitoring shifts in the LSPR wavelength [137]. LSPR sensing platforms consist of either metallic nanoparticles (e.g., nanospheres, nanorods, nanostars), suspended in solution or deposited on a solid support, or micro- and nano- fabricated metallic structures arrays on a solid support (e.g., nanopillar array) [138]. The LSPR peak wavelength can be tuned corresponding to the desired application by modifying the size, shape and material of the nanostructures, which represents an advantage for sensor development [137]. Thanks to the improvement in nanofabrication, various LSPR-based nanosensors have been developed for diverse applications including the detection of various biomolecules such as DNA, disease biomarkers, hormones, amino acids etc. [137,139,140,141], and different chemical compounds such as inorganic gases [142,143] and VOCs [2,144,145,146].

In the present review, we will exclusively focus on propagating SPR-based sensor developed for the detection of VOCs. In the literature, a considerable number of reviews and articles that describe and explain the theory behind this phenomenon (propagating SPR) as well as its application for sensor devices can be found [121,147,148,149,150,151,152,153,154].

In the following sections, we will first present a brief theoretical overview of propagating SPR. Then, we will make a comprehensive review of the progress made in the development of gas sensors and electronic noses that employ this technique. In particular, the different systems will be classified based on their coupling configuration.

### 3.1. The Theory of Propagating SPR

Let us consider a semi-infinite metal with a frequency dependent complex permittivity or dielectric function $\varepsilon_{m}$ and a semi-infinite dielectric with a permittivity $\varepsilon_{d}$, separated by a planar interface. The solution of Maxwell’s equations under appropriate boundary conditions suggests that s-polarized surface oscillations cannot be supported by this type of interface. Consequently, SPWs are transverse-magnetic (TM) or p-polarized waves, i.e., their magnetic field vector is parallel to the interface and perpendicular to the propagation direction [121,154]. Moreover, the existence of surface plasmon requires that the real part of $\varepsilon_{m}$ is negative and its absolute value is greater than $\varepsilon_{d}$. At optical wavelength (visible and near infrared), this condition is satisfied for various metals including gold which is commonly used for sensor applications [153]. From the analysis of Maxwell’s equations, it is also possible to derive the frequency dependent wavevector also called the dispersion relation or propagation constant of the SPW on a smooth surface that is given by [151,155]:

(1) $$k_{SPP}=\frac{\omega }{c}\sqrt{\frac{\varepsilon_{d}\varepsilon_{m}}{\varepsilon_{d}+\varepsilon_{m}}}$$

where *ω*/*c* is the free space wave vector of an optical wave.

The propagation of SPWs along the interface undergoes strong attenuation due to high Ohmic losses in the metal which, consequently, limits the propagation length [149]. This damping is associated with the imaginary part of the wavevector that depends on the metal’s permittivity at the oscillation frequency of the SPW [121,149]. The propagation length along the interface is a few microns or even a few tens of microns depending on the metal and the excitation wavelength used [147]. This length can be expressed as [149]:

(2) $$L_{SPP}=\frac{1}{\left|2Im\left\{k_{SPP}\right\}\right|}$$

**Figure 4:**
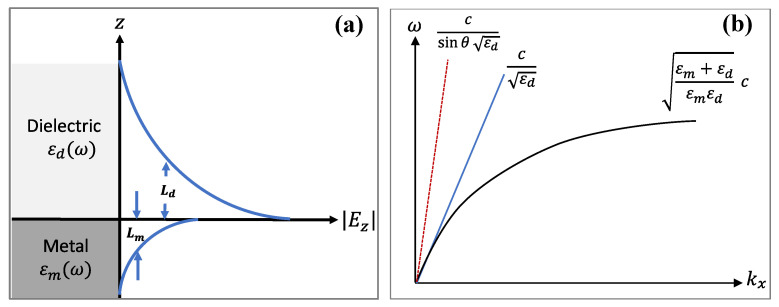
(**a**) Distribution of the electromagnetic field of the surface plasmon polaritons (SPPs) along the *z*-axis (perpendicular to the surface), the intensity of this field is maximum at the surface and decays exponentially away from it. With *L_d_* and *L_m_* the penetration depth in the dielectric and the metal, respectively. (**b**) Dispersion curve of: free photons propagating in a dielectric (blue solid line), x-component of free photons propagating in a dielectric (red dashed line) and SPPs (black solid line).

Confined at the vicinity of the interface, the electromagnetic field associated with the wave decays evanescently into the metal and the dielectric. However, as shown in Figure 4a the distribution of this field is asymmetric and mostly concentrated in the dielectric [148]. This disparity in the penetration depth is due to the fact that the dielectric constant of the metal is greater than that of the dielectric. The field decay from the surface in the adjacent medium is determined by the dispersion relations of the SPW in the direction perpendicular to the interface (i.e., in the dielectric *k_zd_* and in the metal *k_zm_*) [155]:

(3) $$k_{z}=\left\{\begin{array}{c} \frac{\omega }{c}\sqrt{\frac{\varepsilon_{d}^{2}}{\varepsilon_{d}+\varepsilon_{m}}}\;\;\;\;\;\;\;\;\;\;\;\;\;\;\;in\;dielectric;\;\\ \frac{\omega }{c}\sqrt{\frac{\varepsilon_{m}^{2}}{\varepsilon_{d}+\varepsilon_{m}}}\;\;\;\;\;\;\;\;\;\;\;\;\;\;\;in\;metal. \end{array}\right.$$

The decay length also called penetration depth or skin depth of the SPW in the adjacent medium corresponds to the distance from the interface at which the intensity of the field falls to 1/e of its maximum value [148,155]. This value can be expressed as [155]:

(4) $$L_{zi}=\frac{1}{\left|Im\left\{k_{zi}\right\}\right|}\;\mathrm{with}\;i=\mathrm{metal}\;\mathrm{or}\;\mathrm{dielectric}$$

To give an order of magnitude, the penetration depth is a few hundred nm (~200 nm) in the dielectric and a few tens of nm (~25 nm) in the metal [147].

The excitation of surface plasmons or the generation of SPWs at the planar interface requires special configurations. Indeed, for the same frequency, the propagation constant (the wavevector) of the surface plasmon at the metal-dielectric interface (black solid line) is higher than the wavevector of photons in the dielectric (blue solid line) (Figure 4b). This mismatch has two consequences. First, the SPPs cannot radiate in light, and are bound to the surface. Second, they cannot be directly coupled or excited by a conventional light illuminating the metal/dielectric interface. Attenuated total reflection (ATR) or diffraction endows the excitation wave with additional momentum to overcome the mismatch and excite SPPs. In practice, this can be achieved using different coupling systems (couplers) such as prim, waveguide and grating couplers [121,147,148,149]. The excitation of the SPPs manifests itself by a resonant transfer or absorption of the incident light energy resulting in SPR.

As mentioned earlier, SPR is extensively used as transduction technique for optical sensor development and enables the detection of analytes by monitoring changes in the refractive index ($n_{d}$) or permittivity ($\varepsilon_{d}\;with\;\varepsilon_{d}=n_{d}^{2}$) of the dielectric where the sensing material is deposited. Indeed, since the electromagnetic field of the SPWs is mostly concentrated in this medium, the propagation constant of the wave is strongly affected by its optical properties, namely, its refractive index. The characteristics of the exciting light (i.e., its intensity and phase), are altered upon the interaction with the SPPs and, thus, variations in these parameters can be correlated with changes in the propagation constant of the SPWs and thus the refractive index of the dielectric. In other words, binding-induced modulation in the refractive index at the sensor surface and, consequently, the propagation constant of the SPWs can be detected by measuring changes in the output light properties. Finally, it is worth noting that, since the penetration depth of the field in the dielectric is few hundreds of nm (~200 nm), the SPR can only detect binding events taking place below this limit.

In the following parts, we present the different coupling strategies and review the various studies that employed SPR for the detection of odorant molecules.

### 3.2. Prism Coupler-Based Sensors

**Figure 5:**
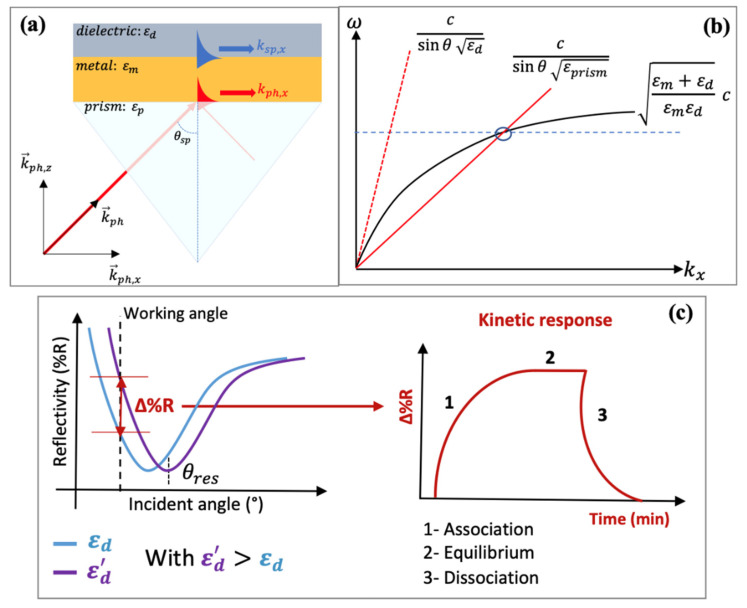
(**a**) Excitation of SPPs by prism coupling in Kretschmann configuration. (**b**) Dispersion curve of: x-component of free photons propagating in a dielectric (red dashed line), x-component of free photons propagating in a prism (red solid line), SPPs (black solid line). (**c**) Intensity interrogation principle.

The excitation of SPPs via ATR and prism coupler was first demonstrated by Otto then by Kretschmann and Raether. The Kretschmann configuration is the most commonly used method. This configuration consists of a thin metal film usually gold (about 50 nm thick) deposited on the surface of a prism on top of which the sensitive material is deposited. As shown in Figure 5a, to provoke the coupling, the prism is illuminated with a p-polarized light wave (since the SPW are p-polarized) at an incident angle greater than the critical angle. When the light reaches the prism-metal interface, it is totally internally reflected and an evanescent photon wave is generated at the interface [147]. The high refractive index or permittivity $\varepsilon_{p}$ of the glass prism allows to enhance the momentum or wavevector of the evanescent wave that can thus excite the SPPs [156]. Resonance occurs when the in-plane component of the incident light (photon) wave vector $k_{ph,x}$ (red solid line), which corresponds to the propagation constant of the evanescent wave, matches that of the SPWs. Consequently, a transfer of energy from the incident light to SPWs occurs and is manifested by a sharp dip in the intensity of the reflected light. To satisfy the matching conditions, the angle of incidence or the wavelength of the exiting light can be adjusted since the propagation constant of the evanescent wave is dependent on these parameters. The terms resonance angle and resonance wavelength correspond to values of incident angle and wavelength at which almost 100% efficient coupling and energy transfer are achieved [156]. The resonance condition can be expressed as [121]:

(5) $$k_{ph,x}=\frac{\omega }{c}\;\sqrt{\varepsilon_{p}}\sin\theta =Re\left\{k_{spp}\right\}$$

The same resonant conditions apply for the Otto configuration. The only difference is that, in this configuration the metal film is separated by a small gap from the surface of the prism [129].

In practice, for sensing applications, the sensitive materials are deposited on top of the metal layer, which allows to customize the sensitivity and selectivity of the sensor. Four main measurement methodologies are employed to detect the kinetic interaction of target molecules with the sensitive materials: intensity interrogation, spectral or wavelength interrogation, angular interrogation and finally phase interrogation [157].

In the first method (namely intensity interrogation), variations in the intensity of the reflected light are monitored over time at a fixed wavelength (i.e., using a monochromatic light source) and fixed incident angle (known as the working angle) (Figure 5). The working angle is usually chosen close to the resonance angle ($\theta_{res}$) where small variations in $\theta_{res}$ caused by modulation of the surface refractive index will result in large shifts in the intensity of the reflected light. On the other hand, for spectral/wavelength interrogation, a broadband or polychromatic light source is used to excite the SPPs at a fixed incident angle and modulations of the resonance wavelength are monitored. Conversely, in the case of angular interrogation, variations in the resonance angle are measured at a fixed wavelength. Finally, for phase interrogation, shifts in the relative phase difference between p- and s-polarization components are monitored at a specific wavelength and angle. This last interrogation technique offers the highest sensitivity but suffers from a narrow dynamic detection range [158]. The different interrogation methods and especially intensity interrogation allow for simultaneous monitoring of binding events occurring on multiple sensors which is particularly beneficial for electronic nose systems. This multiplexing technique is called surface plasmon resonance imaging (SPR imaging) [159].

Many gas sensors and eN systems can be found in the literature based on this configuration and using a large diversity of sensitive materials including biological elements (e.g., olfactory receptors, odorant binding proteins and peptides) and chemical elements (e.g., polymers and calixarenes). The different systems can be classified into two categories depending on the detection medium, i.e., in the liquid or in gas phase.

#### 3.2.1. Detection of VOCs in Liquid Phase

Prism coupler-based SPR has been widely employed to develop biosensors/biochips for the analysis of large biological molecules. However, it is often considered unsuitable or limited for the analysis of low weight molecules such as VOCs (molecular mass < 300 Da) in the liquid phase. To overcome this limitation, it is essential to couple the optical transduction systems with appropriate sensitive materials in order to generate detectable signals upon their interaction with VOCs. Different biological sensing materials (ORs, OBPs, etc.) have been used for such applications. Very often, signal amplification strategies are needed to obtain reliable SPR signals, which will be highlighted in this review.

Selected and improved by natural evolution, olfactory receptors are very attractive candidates. Since their identification and isolation by Buck and Axel, these proteins have been extensively studied [10]. Great research efforts has been made to deorphanize these receptors [160] and improve their large-scale production that was found to be challenging in some early works [161,162]. The use of OR as sensing materials for the development of olfactory biosensor and eNs presents many assets including high sensitivity and selectivity. In addition, they can be genetically modified to facilitate their purification and immobilization. However, being transmembrane proteins, the presence of a lipid bilayer environment is crucial to maintain their three-dimensional structure and retain their activity when immobilized on sensor chips. This task has been a major drawback and challenge for the development of OR-based olfactory biosensor. Nevertheless, several ingenious strategies have been employed to provide the lipidic environment such as the use of plasma membrane fractions, nanovesicles and nanodiscs [26]. Consequently, OR-based sensors were proven to be effective for the detection of VOCs using different transduction systems including QCM [92], FET [163], electrochemical [71].

**Figure 6:**
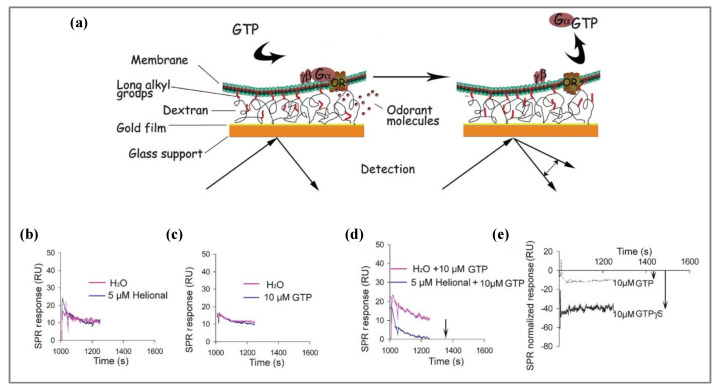
(**a**) BIAcore sensor chip L1 functionalized with nanosomes. No surface plasmon resonance (SPR) response was observed when nanosomes were stimulated either with odorant alone (**b**), or guanosine-5′-triphosphate (GTP) alone (**c**), as compared to the control stimulated with water. The SPR signal was only observed when odorant and GTP were injected simultaneously (**d**). The signal relative to the release of the G_α_ subunit can be further enhanced four-fold by replacing GTP by GTPγS (**e**) [164].

SPR platforms have also been associated with ORs. Pajot-Augy’s group [164] demonstrated the possibility of using mammalian OR as sensing elements for highly sensitive olfactory biosensors. In their study, they first co-expressed rat ORI7 and human OR17-40 and their associated G_αolf_ subunit in yeast cells. To maintain their structure, the ORs were encompassed in membrane fractions that formed nanosomes with a diameter of approximately 50 nm. The nanosomes were then immobilized on a Biacore sensor chip L1, which consisted of a gold-coated glass support functionalized by a covalently linked carboxylated dextran polymer hydrogel grafted with long alkyl chains (Figure 6). Nanosomes were effectively bound by those alkyl anchors. A BIAcore 3000 was used to perform measurements. This type of setup allows the measurement of resonance angle shifts and consists of a near-infrared LED light source for SPR excitation and a linear array of light sensitive diodes to monitor the reflected light. As reported, no SPR signal was observed when VOCs were injected alone due to poor signal/noise ratio. To solve the problem, an indirect ingenious amplification strategy was designed. It consists of taking advantage of the presence of G_αolf_ anchored to the nanosomes to monitor receptor activation by an odorant ligand, through the desorption of G_αolf_ subunit from the lipidic bilayer. In such a way, when a target odorant binds to the OR, the subunit is activated and then desorbs from the lipidic membrane, resulting in a much stronger SPR signal, as illustrated in Figure 6. To trigger this mechanism, VOCs were injected in the presence of guanosine-5′-triphosphate (GTP). The study demonstrated that ORs retained their functionality in membrane fractions even after immobilization and the obtained olfactory biosensor exhibited high sensitivity and selectivity. The sensor chip kept the same activity level for up to eight injection cycles.

In a complementary study [165], using this SPR sensing strategy, they investigated the molecular mechanisms underlying odorant detection, in particular, the role of OBPs in the dynamic interactions between OR and odorant ligands. They showed that OBPs play an active role in preserving the conformation and activity of OR especially at high odorant concentration. This finding revealed another role of OBPs in olfaction, in addition to their role in transporting odorants through the olfactory mucus. Importantly, their study showed that SPR-based olfactory biosensors can be used not only for the analysis of odorant molecules, but also for the investigation of basic biologically relevant questions in olfaction.

**Figure 7:**
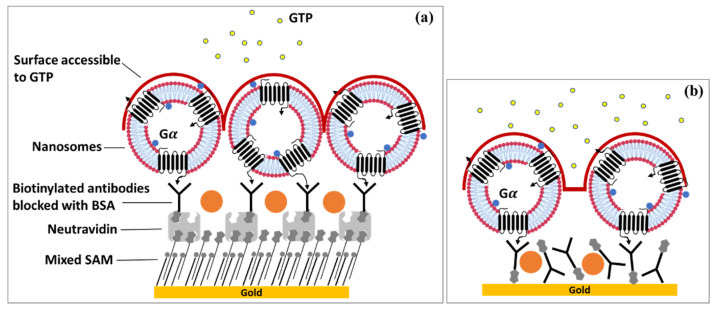
Schemes of the two-surface chemistry employed for the immobilization of olfactory receptors (ORs) in the nanosomes, which were specifically captured via anti-cmyc antibody attached to the gold-coated substrate in an orientated (**a**) or random way (**b**).

Furthermore, in collaboration with Jaffrezic-Renault’s team, they demonstrated the importance of the surface chemistry on the performance of the system [166]. Human OR17-40 modified with a cmyc tag on the N-terminus and its G_αolf_ subunit were co-expressed in yeast cells (*S. cerevisiae*). The receptors carried by nanosomes were attached to the sensor chip through specific antibody-directed immobilization using Anti-cmyc monoclonal antibodies. Two strategies involving different biofilm architectures were explored: one with controlled antibody orientation and the other with random orientation, as illustrated in Figure 7. A Kretschmann-type SPR spectrometer NanoSPR-6 with two optical channels and a diode light source (650 nm wavelength) was used to perform the study. The response of the system was measured in terms of resonance angle modulation. The setup included a double channel Teflon flow cell that allowed signal acquisition in both custom and differential modes (delta between working and reference channels). They showed that the density of nanosomes and the multilayer bulk thickness are crucial factors for the performance of the olfactory biosensor. The biofilms prepared following the first surface chemistry strategy had higher thickness and nanosome density. However, the corresponding olfactory biosensor exhibited a lower sensitivity for the target odorant molecules compared to the OS based on the second surface chemistry. Indeed, the second strategy provided biofilms with lower thickness and higher porosity that allowed a better accessibility of G_αolf_ to GTPγS, and thus, increased sensitivity.

Another strategy to exploit the potential of OR for sensing applications is to use so-called artificial olfactory cells, which are genetically modified cells that express olfactory receptors. Park’s team developed a sensitive and selective SPR-based olfactory biosensor using whole cells expressing olfactory receptors ORI7 as sensitive materials [167]. The cells were attached to a gold-coated glass slide using poly-D-lysine. The slide was put into optical contact with a prism using a refractive index matching fluid. A p-polarized laser light with a wavelength of 670 nm was used as the probe beam. Thanks to a photodiode detector, variations in the reflected light intensity were monitored as a response to analytes.

**Figure 8:**
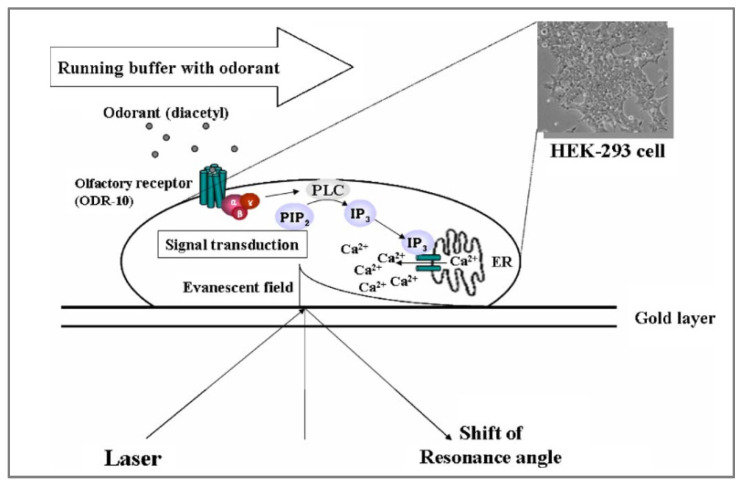
Principle of cell-based measurement of odorant molecules using SPR. An olfactory cell expressing OR was adhered to the gold surface of the sensor chip, and activated by odorant molecule diacetyl. The specific binding of diacetyl to the OR triggered the G protein transduction cascade inside the cell and thus an SPR signal [168].

In this system, the SPR signal was not directly ascribed to the conformational change of the OR or to the desorption of the G_α_ subunit. In fact, the olfactory receptors expressed on the surface of the cell were not in the detectable range of the SPR (approximately 200 nm above the gold surface), since the size of the cell was several micrometers. However, the G-protein transduction cascade induced by odorant binding generated changes in the intracellular components, mainly with an increase in Ca^2+^ ions. Such changes generated a variation in the local refractive index consequently leading to an SPR signal. In a previous study [168], the group had already demonstrated the feasibility and effectiveness of such a system (i.e., an SPR-based sensor with artificial olfactory cells expressing OR) for the detection of odorants (Figure 8).

**Figure 9:**
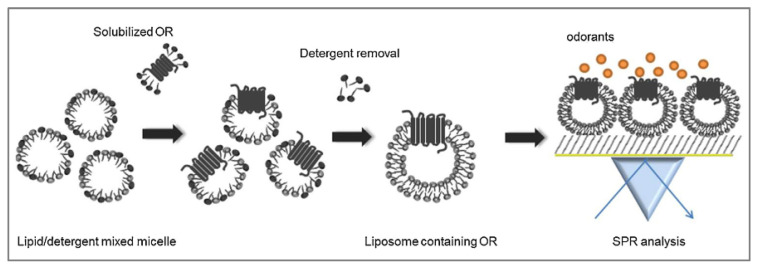
Schematic diagram of reconstitution of OR and SPR analysis. The partially purified OR was reconstituted using lipid/detergent mixed micelle and immobilized on the gold surface of SPR to detect the odorant binding [169].

Although the cell-based olfactory biosensor is very interesting, it is limited by the short lifetime of the sensitive materials. In addition, the system is easily influenced by environmental conditions. Therefore, in another work [169], Park’s team explored a different strategy to provide a natural lipidic environment to maintain the stability and biological function of ORs and that is to use liposomes (Figure 9). They controlled the size of the liposome to 40–50 nm, making them fall within the detectable range of the SPR. The liposomes were then immobilized on the poly-D-lysine-coated SPR sensor chip. Their study demonstrated that the reconstituted ORs carried by liposomes were effective sensitive materials for odorant detection.

In a similar work, Sanmartí-Espinal et al. [170] prepared nanovesicles from yeast membranes, with a size of about ~100 nm in diameter, to carry ORs as sensitive materials. Their SPR-based olfactory biosensor had good selectivity. Based on the SPR signal, they even tried to quantify the number of odorants that interacted with a given olfactory receptor.

In addition to ORs, odorant binding proteins also have great potential as sensitive materials in the field of olfactory biosensors. OBPs are small proteins (~20 kDa) highly concentrated in the nasal mucus of vertebrates [171] and in the sensory lymph of insects [172]. Vertebrate OBPs belong to the lipocalin family, characterized by β-barrel structure with eight antiparallel β-sheets that enclose a hydrophobic binding cavity for odorants also known as calyx [173]. Thanks to their binding pocket, OBPs can reversibly bind odorant with micromolar dissociation constant and a broad affinity spectrum (i.e., can interact with different chemical classes) [173]. These proteins are thought to act as shuttles that facilitate the transport and diffusion of hydrophobic odorants across the aqueous mucus to reach the olfactory receptors [174].

Unlike ORs, OBPs are soluble proteins, and thus, do not require a lipidic environment. This also facilitates their large-scale production and purification. They exhibit good stability to high temperature and pH variations, as well as low susceptibility to proteolytic degradation [27]. Moreover, they have a broad specificity and can be genetically modified to tailor their binding properties or facilitate their immobilization. Despite their high stability, maintaining the activity of these proteins over time after their immobilization on the sensor chip and/or after exposure to VOCs is challenging especially in a dry working environment. Nevertheless, many studies have largely investigated the suitability of these sensitive materials for the development of olfactory biosensors and eNs. Indeed, OBPs have been coupled to different transduction platforms (e.g., SAW [175], FET [176]) and their performance were evaluated in both liquid and gas phase [26].

**Figure 10:**
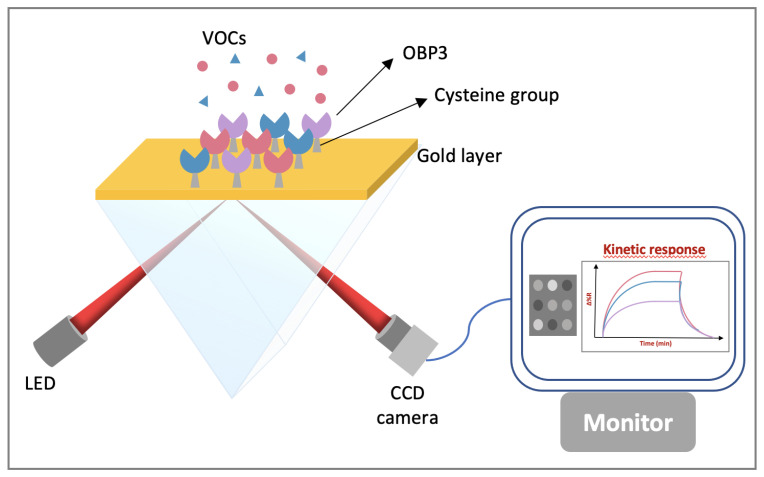
Schematic representation of the SPR-based olfactory biosensor with odorant binding proteins (OBPs) as sensing elements. The three rat OBP3 mutants were immobilized on the gold surface of a prism and their interaction with volatile organic compounds (VOCs) was monitored by SPR imaging.

Recently, our team successfully demonstrated the feasibility of a SPR-based OS with OBPs as sensing elements [177]. For that study, three rat OBP3 derivatives with customized binding properties were designed and produced, including OBP3-w, OBP3-a and OBP3-c. The first protein corresponded to the wild type form while the two others were genetically modified mutants. Thanks to site-directed mutagenesis, the binding affinities of the OBPs were customized by varying certain amino acid residues of their binding site. OBP3-a was tuned to have good affinity for aldehydes by introduction of a lysyl residue, while OBP3-c was modified with bulky amino acids to block the binding pocket. Consequently, it could no longer interact with VOCs and was used as negative control. The recombinant proteins were all expressed in *E. coli*. They were immobilized by self-assembly on gold-coated prism by means of a cysteine group that was introduced to their N-terminus, located on the opposite side of the binding cavity. This functionalization strategy allowed easy and orientation-controlled protein immobilization with the OBP at the vicinity of the gold surface. The SPR measurements were performed using a commercial SPR imaging apparatus (SRRiPlex from Horiba). The microarray was illuminated with p-polarized light at 663 nm wavelength. The intensity modulation of the reflected light at a fixed working angle of all the sensors was monitored simultaneously thanks to a CCD camera upon addition of VOCs (Figure 10).

The obtained SPR-based olfactory biosensor had a very low detection limit (DL), e.g., 200 pM for the odorant β-ionone. This result is among the lowest DL reported in the literature. Moreover, the SPR system was able to detect odorants with a molecular weight of 100 g/mol (hexanal) which is lower than DL in mass commonly admitted for commercial SPR imaging, namely, 200 g/mol. Indeed, the intensity of the SPR signal obtained could not be explained solely by the increase in mass after the binding of VOCs on the chip. It is very likely that the binding of VOCs to OBPs induced a conformational change, which led to a variation of the local refractive index with amplified SPR imaging signals. This was possible thanks to our functionalization strategy that enabled the immobilization of the OBPs at the vicinity of the gold surface. Moreover, at low VOC concentration, the olfactory biosensor exhibited an extremely high selectivity with great potential for trace VOC detection.

Biomaterials unrelated to the olfactory system were also used as sensitive materials. Dung et al. developed an efficient SPR-based olfactory biosensor for the detection of toluene using the toluene binding domain (TBD) [178]. TBD belongs to the TodS protein present in the bacterium *Pseudomonas putida*. In this study, a direct immobilization strategy was also employed by introducing three cysteine residues to the N-terminus of the TBD protein. This allows, on the one hand, to control the protein orientation to ensure good accessibility of the binding pocket, on the other hand, to detect SPR signal induced by the conformational change of TBD upon toluene binding. Shifts in reflected light intensity were monitored by a photodiode receptor as a response to analytes. The TBD-based olfactory biosensor showed not only good sensitivity for the target VOC, with DL at 15.62 µM, but also high specificity, with no response for other aromatic hydrocarbons, such as p-xylene and benzene.

The Table 1 summarizes the conditions for VOCs detection of SPR-based olfactory biosensors and their performances in liquid phase.

#### 3.2.2. Detection of VOCs in Gas Phase

The first studies showing the feasibility of prism coupler-based SPR for gas detection date back to the early 1980s [119,179]. However, very few examples were reported in the literature before 2000 [180,181,182,183,184]. These systems were limited in terms of sensitivity and selectivity based on only one or few sensitive chemical layers. Since 2000, an increasing number of articles can be found in the literature using both biological and organic sensitive materials [185,186,187,188,189,190,191,192,193,194,195,196,197,198,199,200,201,202,203,204]. It has been demonstrated that SPR is very effective for sensing VOCs in the gas phase. In fact, when using air as the analysis medium, the detection noise remains relatively low thanks to the low optical index of this medium. Consequently, the binding of the small VOCs can generate reliable SPR signal with very high signal/noise ratio.

For the development of SPR-based olfactory biosensors and eNs for VOC detection in the gas phase, the use of biomolecules such as ORs and OBPs as sensitive materials is limited by their stability under such conditions. Their peptide analogues are particularly interesting alternatives. Indeed, peptides, and in particular, short ones, do not require specific conditions (i.e., humidity, temperature, phospholipidic matrix) to maintain their activity. Moreover, they are much easier to produce and immobilize onto a sensing platform.

**Figure 11:**
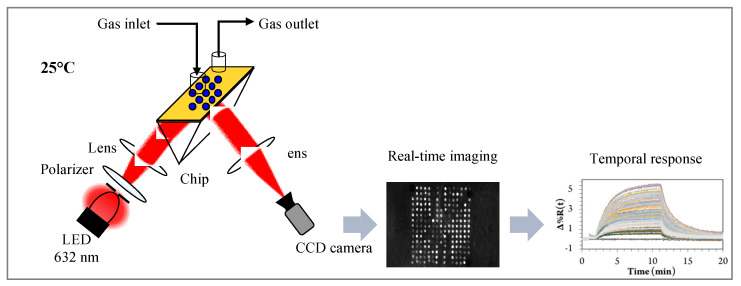
Schematic presentation of the home-made SPR imaging setup.

Recently, our group developed an innovative optoelectronic nose using biomimetic peptides based on SPR imaging for the detection of VOCs in the gas phase [185]. For this purpose, a homemade SPR imaging system based on the Kretschmann configuration was constructed, shown in Figure 11. A polarized LED light beam with a 632 nm wavelength was used to excite SPs and a 16-bit CDD camera was used to simultaneously monitor the reflectivity of all the sensors on the chip in real-time. Variation in the reflectivity at a fixed working angle was measured over time upon the exposure of the sensor microarray to VOCs, providing a temporal response.

Such an SPR imaging system is very promising for the development of eN. First, a chip consisting of a large sensor array can be easily prepared and used. The number of sensors is only limited by the resolution of the microarray printing of the sensitive materials. Second, thanks to the imaging mode, the interactions between VOCs and all sensors can be simultaneously monitored using the same instrument. Finally, SPR imaging can provide temporal responses with additional kinetic information compared to a simple equilibrium response obtained with most of the existing eNs.

The peptides were all terminated by a cysteine for their direct immobilization on the gold surface of prism. Thanks to their diverse physicochemical properties and cross-reactivity for VOCs, the obtained eN was found to be very effective in sensing VOCs of different families. In particular, it exhibited extremely high selectivity, capable of discriminating between VOCs differing by a single carbon atom. Additionally, it showed good repeatability and stability under repeated use and prolonged storage.

In order to improve the performance of our eN, in another study [186], we investigated the influence of the wavelength of the LED on the sensitivity of the system by combining numerical simulations with experimental validation. The results showed that the angular sensitivity increased with the wavelength but the angular linearity range decreased due to the narrowing of the plasmon resonance curve at high wavelength. Therefore, a compromise must be made to choose the optimal wavelength depending on the study purposes. Under optimal conditions, the detection limits of our eN reach low parts per billion (ppb) range for VOCs such as 1-butanol.

Furthermore, we investigated the optical contributions to the sensitivity of the SPR imaging [187]. For this, an original characterization method, which was independent of the carrier gas, was established for the SPR prism sensitivity based on pressure jumps [205]. In this work, the impact of different adhesive layer (Cr, Ti) as well as surface topography on the system sensitivity was evaluated. It was found that even though slightly higher sensitivities were theoretically achieved using Ti/Au prism, Cr/Au prisms were more suitable for eN applications since they showed lower sensitivity variabilities, noise, and signal drift due to better adhesive properties. Furthermore, the sensitivity loss due to Au grain-related SPP damping was fully characterized and numerically validated to be free from additional fitting parameters. The adsorption of water vapor was later characterized for such Au surfaces to understand humidity related effects on the eN system. Finally, our study showed that prism sensitivity decreased with increasing temperature [206].

In order to diversify the sensitive materials for eN development, in collaboration with Compagnone’s team [191], we tested six novel penta-peptides and nine hairpin DNA selected by virtual screening. Thanks to the complementarity of their binding properties, the obtained eN was able to discriminate not only between VOCs of different chemical families, but also VOCs from the same family with only 1-carbon difference such as 1-butanol and 1-pentanol.

**Figure 12:**
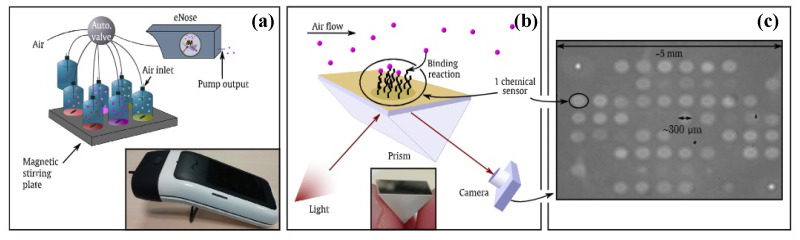
(**a**) Portable NeOse Pro and the experimental set-up for VOC sampling, (**b**) working principal and (**c**) raw image of the prism surface with each spot corresponding to a sensor [188].

Considering the outstanding potential of our eN system and its great ability to detect and discriminate VOCs, a miniaturized version, called NeOse Pro, was further developed by the company Aryballe. Using the same biomimetic peptide-based chip, Maho et al. [188] demonstrated that NeOse Pro was even able to discriminate between two chiral forms ((*R*) and (*S*)) of Carvone and Limonene (Figure 12). Such performance is exceptional for eN system.

**Figure 13:**
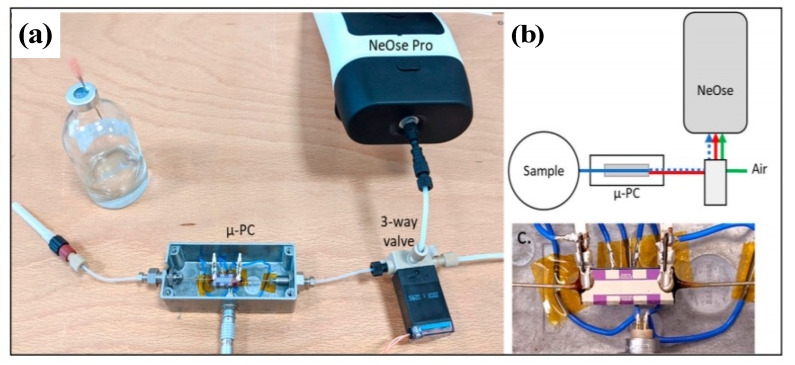
NeOse Pro and µ-preconcentration system coupling. (**a**) Experimental setup, with the sample vial. (**b**) Schematic view of the NeOse Pro/micro preconcentrator (µPC) system and (**c**) View of the preconcentration chip on the metalized side [189].

NeOse Pro is a very promising tool for field analysis, although, as with most eNs, its use for the headspace analysis of highly humid samples remains a challenge, since its performance may be deteriorated by the presence of a high background signal generated by water vapor from aqueous samples. Slimani et al. [189] have tackled this issue by using a miniaturized silicon preconcentrator packed with hydrophobic adsorbent coupled to the NeOse Pro (Figure 13). As a result, the eN showed not only a great improvement in the detection limit (lowered by 125-fold) for target VOCs, but also an enhancement in the discrimination ability demonstrated by the analysis of eight different flavored waters.

In a recent study, Fournel et al. [190] compared the performance of the NeOse Pro with human olfaction. They found that the responses of the eN were not a mere reflection of the chemical space of odorants, but rather, that semantic dimensions were also prominent, similar to natural olfaction.

Besides biomolecules, chemical sensitive materials such as cavitands (calixarenes, cyclodextrins) were also used for the detection of gaseous VOCs with prism coupler-based SPR. They are very interesting for trapping VOCs thanks to their molecular structures with cavities, whose sizes, shape and physicochemical properties can be tuned using a wide variety of functional groups.

Daly et al. [192] ingeniously designed new cavitands containing a carboxylic acid group at the upper rim of the cavity for the detection of organophosphorus vapors, and in particular, the sarin nerve gas stimulant dimethylmethylphosphonate (DMMP). The formation of a hydrogen bond between the COOH moieties and P = O group of DMMP was expected. Two different cavitands with four alkyl feet (five carbons long) were produced and studied. Both molecules had similar cavities but with the carboxylic acid group pointing either out of or into the cavity. Their sensitivity to DMMP was compared with that of fluoropolyol, a commonly used polymeric sensing layer for DMMP detection. Cavitands and fluoropolyol layers were deposited on gold-coated glass slide by spin coating and Langmuir-Blodgett technique for comparison. Both techniques allow for the preparation of uniform and homogeneous thin films with a controlled thickness. To perform measurements, a variable wavelength SPR setup in the Kretschmann configuration was used. The interaction between the DMMP and the sensing layers was monitored by measuring the shift in the SPR wavelength at a fixed incident angle upon the exposure to analytes. The results showed that both cavitand layers exhibited almost the same sensitivity and were able to detect ppb levels of DMMP with a rapid and reversible response. The orientation of the COOH group had no effect on DMMP binding, but had strong impact on water uptake. The cavitand-based gas sensor outperformed the fluoropolyol-based one in terms of DMMP sensitivity and with less interference from water vapor and alcohol. Therefore, such a gas sensor is promising for sensitive and specific detection for nerve gas agents. Moreover, the use of cavitands as sensitive materials for SPR based detection of aromatic vapors was also reported by Feresenbet et al. [193].

In a recent study, Şen et al. [194] worked on the development of gas sensor for the detection of VOCs and in particular acetone using synthesized tetranitro-oxacalix[4]arenes. To perform the study, three nitro-substituted heterocalix[4]arenes were synthesized. Thin films of the three sensing materials were deposited on a substrate by spin coating. Their sensing properties for acetone, chloroform, toluene, ethanol and benzene vapors were evaluated by SPR. A BIOSUPLAR 6 Model spectrometer was used to perform SPR measurements. A p-polarized light with a wavelength of 632.8 nm was used to excite the SP. The intensity of the reflected light at a fixed working angle was recorded by a photodetector as a function of time upon the injection of VOCs. The sensing performance of the three films were investigated at room temperature and the VOCs were carried by dry air to avoid the effect of water vapor. As a result, two of the three thin films showed high sensitivity and selectivity to acetone with a detection limit of 3.8 ppm. The system also exhibited a fast and reproducible response with short recovery times (few seconds).

Other chemical sensitive materials such as polymers were also explored and combined with prism coupler-based SPR for the development of gas sensor. Capan et al. [195] investigated the performance of poly(methylmethacrylate) (PMMA) film as a sensitive material for the detection of BTEX (benzene, toluene, ethylbenzene and m-xylene). PMMA films with different thicknesses were deposited onto gold coated glass substrates by spin coating. The different films were obtained by varying the concentration of the polymer solution and the spin speed. The SPR measurements were performed using a Kretschmann type optical setup and a p-polarized monochromatic light at a wavelength of 633 nm was used to excite the SPs. Optical contact between the substrates and a semicylindrical prism was achieved using an index-matching liquid. Two interrogation methods were used to monitor the response of the system upon VOC injection: modulation in the reflection intensity over time at a fixed working angle and shifts in the resonance angle. As a result, among all the BTEX gases, benzene produced the highest SPR response when exposed to PMMA films. Moreover, the response to the other VOCs was very low which indicated that the gas sensor had high selectivity to benzene. Additionally, the team studied other sensitive materials such as calix-4-resorcinarene films [196] and poly[3-(6-methoxyhexyl)thiophene] derivatives films [197] for sensing BTEX and other VOCs using SPR.

Nanto et al. [198] also used synthetic polymer thin films as sensing membranes for the detection of harmful gases such as ammonia and amines with an SPR-based sensor in the Kretschmann configuration. An LED emitting at a wavelength of 660 nm was used as light source and the reflected light was measured by a CCD camera. The response of the system was measured in terms of modulation of the resonance angle as a function of time upon VOC injection. The sensitivity of two types of polymers was investigated: acrylic acid and styrene. A thin film (several tens of nm) of each polymer was deposited on the gold-coated surface of a prism using plasma chemical vapor deposition (CVD). The response of both membranes was tested against eleven harmful gases: ammonia, acetaldehyde, propionaldehyde, xylene, toluene, trimethylamine, triethylamine, dimethyamine, hormaldehyde, acetic acid and butyl acetate. As a result, the gas sensor with the acrylic acid membrane responded only to the basic gases (i.e., ammonia and amines) with high sensitivity and selectivity. In contrast, the OS with the styrene membrane exhibited a 200 times lower sensitivity. The system with the acrylic acid membrane also exhibited a linear response for ammonia in the range of 50–300 ppm and with an estimated detection limit of several ppm. Finally, the study showed that the thickness of the sensing membrane can be optimized to improve the sensitivity. In another study, using a similar system, Nanto’s team [199] successfully demonstrated the feasibility of multiplexing with a two-channel odor sensor able to simultaneously detect ammonia and acetic acid with high selectivity. The sensor was based on the same SPR setup but with two sensing membrane, namely, acrylic acid and N,N-dimethylacetamide thin films deposited on one chip by CVD. Two channels of the CCD camera were used to monitor the response to VOCs.

To improve the sensitivity of polymer-based gas sensors, one strategy is to introduce nanoparticles (NPs) such as gold NPs. According to the literature the incorporation of Au NPs in SPR sensors could enhance the sensitivity of the device [207]. Indeed, with a rational design, coupling between the localized surface plasmons of the Au NPs and the propagating surface plasmons of the Au substrate may take place, which can result in a larger plasmon angle shift and changes in reflectivity.

Sih et al. [200] developed an SPR-based gas sensor for the detection of alcohol vapors. In the study, the performance of polythiophene (PT) films as a sensing material was compared with that of Au NPs thin films capped with conjugated oligothiophenes. SPR measurements were performed using a Kretschmann configuration setup and a p-polarized light at a wavelength of 632.8 nm was used to excite the SPs. To prepare the chips, the Au NP/oligothiophene (NPOT) film (~60 nm thickness) was electrodeposited on a gold-coated glass slide and the PT film (~7 nm thickness) was deposited by electropolymerization. The response of the sensors was monitored by measuring the shift of the resonance angle. The performance of the two sensitive materials was tested upon exposure to vapors of five solvents: hexanes, toluene, ethanol, methanol, and water. As a result, the PT layer responded to ethanol, methanol and toluene whereas the NPOT film responded exclusively to alcohols. Therefore, there was an improvement in selectivity in incorporating Au NPs. However, in this study no significant improvement in sensor sensitivity was observed.

**Figure 14:**
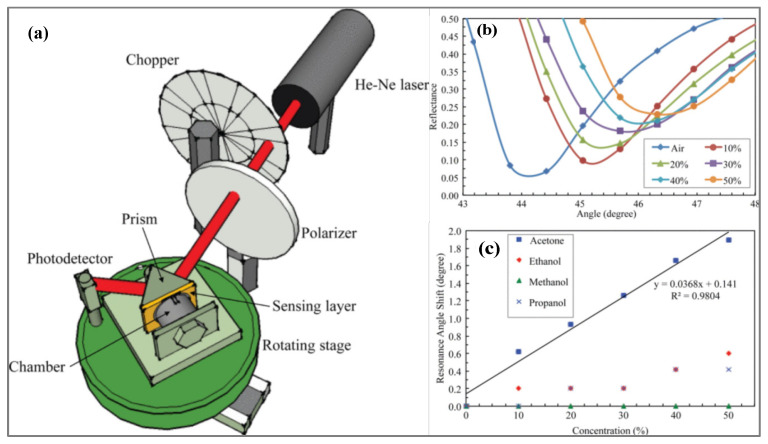
(**a**) SPR setup for detection of hydrocarbon vapor using trilayer Au-rGO/γ-Fe_2_O_3_-Au sensor. (**b**) SPR signals of the acetone vapor detection using the reduced graphene oxide/maghemite (rGO/γ-Fe_2_O_3_) sensing layer. (**c**) its resonance angle shift for increasing concentrations of different hydrocarbon [201].

Another advantage of nanostructures for gas sensor application is the high surface to volume ratio. Alwahib et al. [201] tested the efficiency of a SPR-based OS with a reduced graphene oxide/maghemite (rGO/γ-Fe_2_O_3_) nanocomposite film as sensing layer for hydrocarbon vapor detection. They used a kretschmann-based SPR setup with a helium-neon (He-Ne) laser at 633 nm emission wavelength. A chopper and a polarizer were used to generate the p-polarized excitation beam and a photodetector to monitor the reflected light (Figure 14). Trilayer and bilayer sensing membranes were prepared and compared. The former consisted of a nanocomposite layer (3 nm thick) sandwiched between two gold layers (bottom layer: 37 nm thick, top layer: 2.7 nm thick). For the latter, the rGO/γ-Fe_2_O_3_ film (3 nm thick) was deposited on top of a gold layer (49 nm thick). The sensing membranes were placed on microscope glass slides and then brought into contact with a high index prism using an index-matching liquid. The response of the system, upon the exposure to acetone, ethanol, methanol and propanol, was monitored in terms of modulation of the resonance angle. The SPR signal resulted from the adsorption of hydrocarbon vapors that diffused through the pores of the sensing layer inducing a change of the refractive index. As a result, the trilayer-based gas sensor showed higher sensitivity to acetone compared to the other hydrocarbons. Furthermore, it was more stable and had shorter response time comparing to the bilayer-based gas sensor. The authors concluded that this improvement was due to the presence of the third gold layer, which promotes better interactions.

Apart from the improvements of the SPR sensitivity by the optimization of the optical parameters and the use of NPs as described earlier, other approaches have been proposed in the literature based on active plasmonics to add active functionalities to SPR-based devices. For example, Manera et al. [202] reported a study where magnetic field was used to control the SPR. They compared the sensing performance of a magneto-optical SPR (MO-SPR) sensor with that of a traditional SPR sensor for the detection of alcohol. A home-made setup with the Kretschmann configuration was employed to perform the measurements and a p-polarized light with a wavelength of 632.8 nm was used to excite the surface plasmons. To prepare the MO-SPR sensor, a multilayer of Cr/Au/Co/Au was deposited on a glass substrate. Then, a nanoporous TiO_2_ thin film, used as sensitive material was deposited on top of the multilayer by glancing-angle deposition (GLAD). For comparison, a substrate for classical SPR was also prepared by depositing the TiO_2_ layer on top of a gold-coated glass substrate. Three VOCs were analyzed, including ethanol, methanol and iso-propanol. The MO-SPR based gas sensor exhibited a significant improvement in sensitivity. Furthermore, its sensitivity was also much higher than that of their previous SPR-based gas sensor using TiO_2_ thin films [203] and nanometric polyimide films [204].

The Table 2 summarizes the conditions for VOCs detection of SPR-based artificial olfaction systems and their performances in gas phase.

### 3.3. Wave Guide Coupler-Based Sensors

The fundamental coupling principle using a waveguide is similar to that of the prism, whereby the excitation of surface plasmons is achieved by an evanescent wave generated by ATR. To clarify the terms, an optical fiber is a special type of waveguide, and one that is widely used. Indeed, fiber optics are less expensive than waveguides and have good flexibility, remote sensing capability and other important features presented earlier. The VOC sensors systems that will be presented in the following section will exclusively involve the use of fiber optic SPR (FO-SPR) sensing platforms.

**Figure 15:**
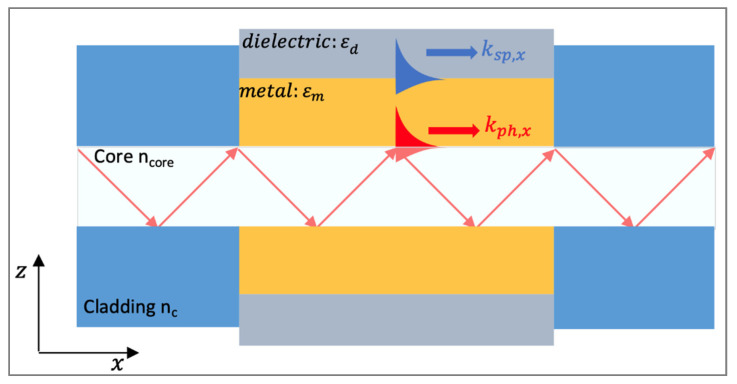
Typical fiber optic SPR (FO-SPR) sensor in transmission configuration.

Fiber optic-based SPR sensors can be elaborated based on either transmission or reflection configuration. A typical fiber optic consists of high refractive index material (the core) sandwiched with a lower refractive index layer (the cladding) which allows light guidance through a succession of total internal reflections (TIRs). In the case of an FO-SPR in transmission configuration, a small region of the optical fiber cladding is removed and replaced by a metal layer where the SPR phenomenon will take place (Figure 15). In the reflection configuration, a thick metal layer deposited at the end of the fiber allows for the SPWs generation and plays the role of a mirror. In both cases, the sensitive materials are deposited on top of the metal layers. As with prism coupling, resonance occurs when the propagation constant of the evanescent wave generated by ATR of the guided mode matches the propagation constant of the SPWs [156]. In SPR-based optical fiber sensors, most of the interrogation methods are based on the detection of loss in the transmitted/reflected light at the resonance. Spectral or wavelength interrogation of the transmitted or the back-reflected light is the most commonly used measurement method. However, fiber-optic sensors based on intensity or phase interrogation have also been reported [208]. Theoretically, the sensitivity of waveguide-based SPR sensors is approximately the same as that of the corresponding ATR configurations [147].

**Figure 16:**
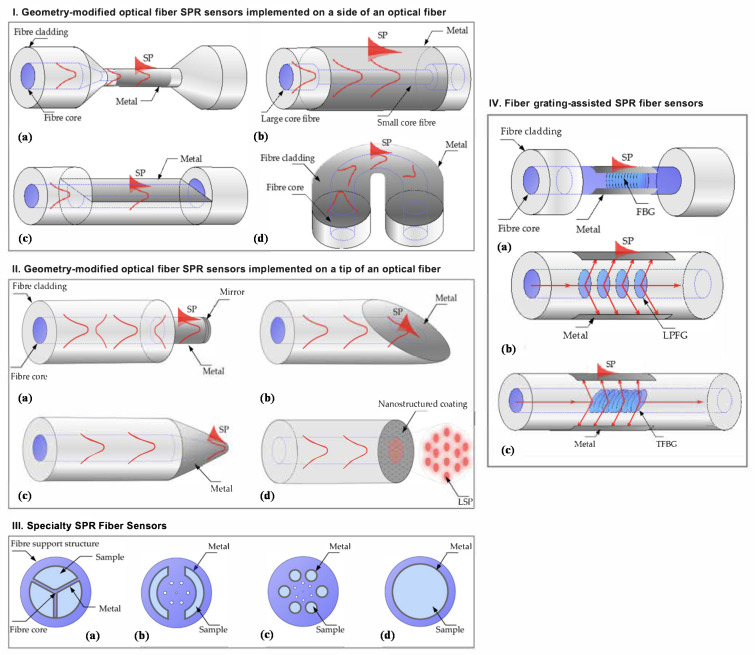
Schematic representation of the different plasmonic fiber-optic sensors **I**: (**a**) Unclad/etched/tapered fiber SPR probe; (**b**) Hetero-core structure; (**c**) Side-polished/D-shaped SPR probe; (**d**) U-shaped SPR probe. **II**: (**a**) Flat fiber tip SPR probe with end mirror; (**b**) Angle polished flat fiber tip SPR sensor; (**c**) Tapered tip SPR probe; (**d**) LSPR fiber tip probe. **III**: (**a**) Wagon-wheel fiber SPR sensor with triangular hole geometry; (**b**) Microstructured optical fiber SPR sensor with crescent-shaped holes; (**c**) Photonic crystal fiber SPR sensor with circular holes; (**d**) Microcapillary fiber SPR sensor geometry. **IV**: (**a**) Etched Fiber Bragg Grating SPR sensor; (**b**) Long Period Fiber Grating SPR sensor; (**c**) Tilted Fiber Bragg Grating SPR sensor [208].

The first fiber optic based SPR sensor with a conventional geometry (as the one presented in Figure 15) and using spectral interrogation as measurement methodology was proposed by Jorgenson et al. [209] in 1993 for a chemical sensing application. Since then, a large number of studies have experimentally and/or theoretically explored diverse geometry-modified single mode or multimode fibers including side and tip implemented FOS, fiber gratings (e.g., long period fiber gratings and tilted fiber Bragg gratings) and specialty fibers [208] (Figure 16). Different plasmonic coatings (e.g., gold, silver) have also been explored. Moreover, configurations involving the excitation of SPPs on continuous thin metallic layers (i.e., propagating SPPs) as well as those involving LSPR phenomena in metallic nanoparticles at visible and near-infrared wavelengths have been reported and reviewed [210].

The effectiveness of these sensors has been extensively investigated for physical (e.g., temperature, humidity), chemical (e.g., pH, gas, VOCs) and biological (e.g., DNA, proteins) sensing applications [156,208,210,211,212,213,214]. In the following section, we will focus on the FO-SPR sensors with different configurations developed for the detection of VOCs. Just like prism-based SPR sensor systems, most of the studies on FO-SPR sensors for the detection of VOCs have been reported after the year 2000 [2,215,216,217,218,219,220,221,222,223,224,225]. Only a few papers were published in the 1990s [226,227].

**Figure 17:**
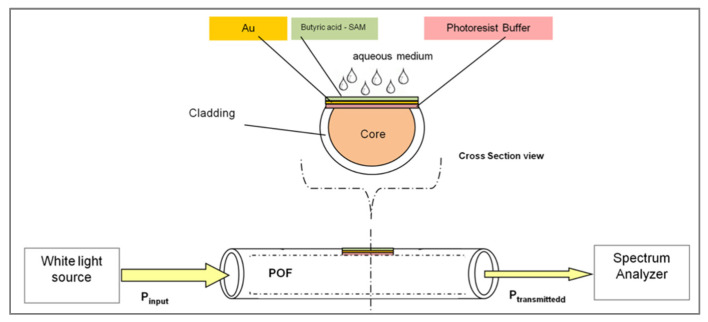
The olfactory biosensor using SPR based plastic optical fiber [215].

With the aim of achieving simple, low-cost and selective detection of aldehydes (known as cytotoxic and carcinogenic compounds) present in the environmental water, Cennamo et al. [215] developed an SPR sensor using plastic optical fiber (POF). To perform the study, butanal was used as the target VOC and porcine OBP (pOBP) as the sensing material. A plastic optical fiber consisting of a PMMA core of 980 μm and a fluorinated polymer cladding of 20 μm was used to elaborate the sensing platform. For that, the cladding of the POF along half the circumference and about 10 mm in length was removed. The exposed core was then coated with a photoresist buffer (1.5 µm thick) on top of which a 60 nm gold layer was deposited (Figure 17). For signal amplification purposes, a competitive assay was designed. For this, instead of OBP, butanal moieties were immobilized on the gold surface of the POF. Then, to test the detection performance, the sensor was exposed to OBPs pre-incubated with/without butanal. Binding events were detected by monitoring variations in the resonance wavelength. A halogen lamp with a wavelength emission range from 360 nm to 1700 nm was used as light source and the transmitted light spectrum was measured using a spectrum analyzer with a detection range of 200 nm to 850 nm. In a first step, the sensor was subjected to OBP (not pre-incubated with butanal), an increasing response was observed for increasing concentration of OBP. This result confirmed that pOBPs bind to the butanal moieties fixed on the chip, which is a prerequisite for the competitive assay. Next, the sensor was exposed to different concentrations of butanal pre-incubated with a fixed concentration of OBP. The results showed that the lower the concentration of butanal (in the pre-incubation solution), the higher the optical signal obtained. Indeed, the lower concentration of butanal resulted in more free OBPs available to bind to butanal moieties on the sensor surface. The obtained olfactory biosensor was able to detect butanal in aqueous solution for concentrations ranging from 20 μM to 1000 μM.

In a previous study [216] the team combined the SPR based POF platform with MIP as sensing material to achieve selective sensing of explosives such as 2,3,6-trinitrotoluene (TNT) in aqueous medium. The system exhibited a detection limit of 5.1 × 10^−5^ M and a sensitivity of 2.7 × 10^4^ nm/M. The authors concluded that despite its limited sensitivity, the sensor was suitable for the detection of TNT with good selectivity. Additionally, the system was easy to prepare and suitable for rapid measurements that did not require any particular skill.

**Figure 18:**
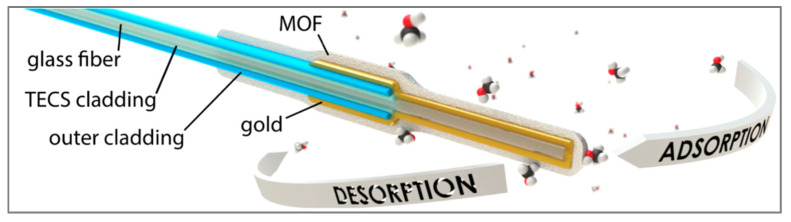
Schematic representation of a metal organic framework FO-SPR probe, not drawn to scale [217].

Vandezande et al. [217], designed a FO-SPR sensor for the detection of alcohol vapors. The sensor consisted of an optical fiber with a diameter of 400 μm, from which the inner technology enhanced clad silica (TECS) cladding and the outer protective cladding had been removed from the end. The exposed glass core was then coated with a 39 nm thick gold layer. Metal organic frameworks (MOFs) and more specifically zeolitic imidazolate framework (ZIF) were used as sensing materials and deposited on top of the gold layer Figure 18. MOFs consist of metal ions or a metal oxide cluster interlinked by polydentate linkers into a crystalline 3D framework. These porous materials have large surface area and tunable pore size, which are attractive features for gas and VOC sensing applications [228]. ZIFs were selected among other MOFs because of their high chemical stability and their small pore sizes. In this study, the sensing ability of two ZIF materials: ZIF-8 and ZIF-93, was explored for the detection of different alcohol vapors including methanol, ethanol, isopropanol, and n-butanol. The response of the system was expressed in terms of changes in the refractive index converted from the SPR response. This made it possible not only to monitor the mass and density changes during layer formation of the ZIFs, but also to investigate sorption behavior of VOCs on these layers. The obtained FO-SPR sensors were able to detect VOCs with ppm concentrations and with a detection limit of 2.5 ppm for methanol. However, a significant drift was observed after extended analysis periods. In this study, the authors claimed that the difference in recognition behavior of the hydrophobic ZIF-8 and more hydrophilic ZIF-93 could be exploited to generate qualitative information regarding the vapor composition.

Gupta’s group published several studies on the detection of VOCs and other odorant molecules using FO-SPR sensors [218,219,220]. In one of these studies [220], they explored the sensing ability of graphene-carbon nanotubes/poly(methyl methacrylate) (GCNT/PMMA) hybrid composites for the detection of methane gas. Their sensitivity and selectivity were compared to that of three other sensing materials including reduced graphene oxide (rGO), carbon nanotubes (CNT), reduced graphene oxide-carbon nanotubes (GCNT). To fabricate different probes, 24 cm long plastic clad silica optical fibers (core diameter 600 μm, numerical aperture 0.4) were used. About 1 cm length of the cladding was removed from the middle portion of the fibers and the uncladded core was coated with a silver layer via thermal evaporation technique. Finally, the sensing materials were deposited on top of the silver by dip coating. To test the performance of the fabricated system, the probe was installed in a gas chamber and a polychromatic light from a tungsten halogen lamp was launched at the input end of the fiber. The spectrum of the transmitted light was recorded with a spectrometer at the other end. The FO-SPR sensors were exposed to different concentrations of methane (ranging from 10 to 100 ppm) and their performance was analyzed in terms of resonance wavelength shift. To evaluate their selectivity, the sensors were exposed to different gases: methane, ammonia, hydrogen sulfide, chlorine, carbon dioxide, hydrogen, and nitrogen. The FO-SPR sensor based on (GCNT/PMMA) hybrid composites showed the best sensitivity and selectivity to methane gas comparing to the three others using rGO, CNT, and GCNT as sensing materials. The authors attributed this performance to the high aspect ratio and the large defect level in the nanocomposite material, which could provide more active sites for VOC adsorption.

**Figure 19:**
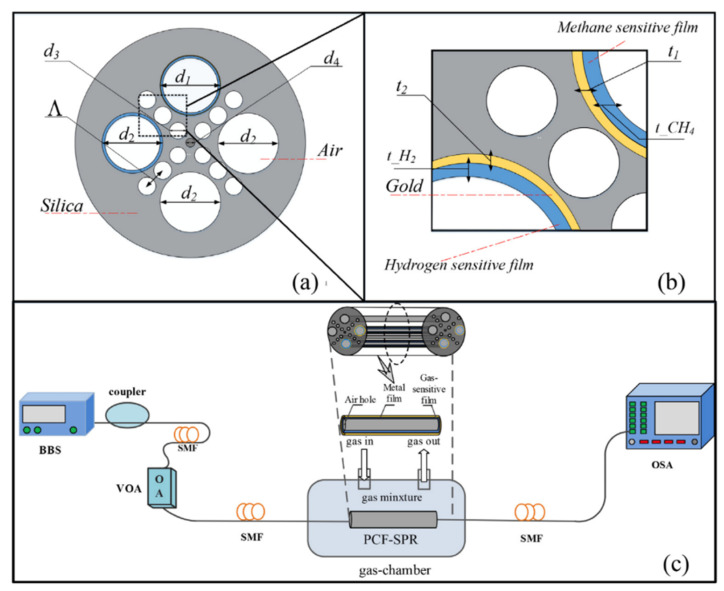
The schematic and cross section of photonic crystal fiber SPR sensor. (**a**,**b**) structural parameters and (**c**) experimental scheme [221].

Photonic crystal fiber (PCF) is a class of optical fiber characterized by a flexible structure design, which presents a unique light controlling capability with light confinement characteristics not achievable using conventional optical fiber. Combined with SPR, PCF can form a very attractive platform for optical sensing. Accordingly, Lui et al. [221] proposed a novel PCF-SPR sensor to detect mixture of methane and hydrogen. As presented in Figure 19, the PCF-SPR sensor consisted of four ultra-large side-holes symmetrically introduced into the cladding layer. These holes allowed to improve the sensitivity to VOCs since the refractive index variation due to concentration change is usually very low. In practice, the two rows of smaller air-holes along the angle of 45° and 135° surrounding the fiber enabled the introduction of the ultra-large side-holes much closer to the fiber core, which, consequently, led to higher sensitivity. The inner surfaces of the left and top ultra-large air-holes were coated with a gold layer on top of which a film of sensing material was deposited. A film of Pd-WO_3_ deposited via the sol-gel scheme was used for the detection of hydrogen. The methane-sensitive film consisted of a kind of ultraviolet curable fluoro-siloxane nanofilm with the inclusion of cryptophane A. It was deposited on the gold layer via a capillary dip-coating technique. The sensing performance and response of the system were characterized by analyzing the confinement loss spectra. As a result, the study showed that using polarization filtering, the concentration of methane and hydrogen in a gas mixture could be accurately measured without interfering with each other. The authors suggested that this approach could be broadened to achieve qualitative identification of multiple gases.

Arasu et al. [222] reported a single mode fiber Bragg grating (FBG)-based FO-SPR sensor coated with graphene oxide (GO) layer for ethanol sensing in an aqueous medium. To fabricate the sensor, a standard single mode FBG with a 9 µm core diameter and 125 µm cladding diameter was used. The polymer coating directly over the Bragg grating was removed. Then, a 45 nm thick gold layer was deposited over the grating area without removing the cladding. Finally, a nanostructured GO layer was put on top of the gold surface by drop-casting technique. A tungsten halogen white light source was employed to generate the input signal and the output light was analyzed by a spectrometer. Wavelength interrogation was used to monitor the response of the system upon the addition of different concentrations of ethanol in water.

In order to make sure that the FBG was effective for SPR sensing without the removal of the cladding layer, the team compared the beam profile of a gold coated FBG to that of a standard gold coated single mode fiber (SMF). The results confirmed that, in contrast to the standard SMF, the FBG was able to scatter the light from the fiber core into the cladding, producing TIR at the cladding-air interface and, thus, an evanescent wave that could be exploited for SPR. They also compared the intensity spectrum of a bare FBG, a gold coated FBG and a gold coated FBG with the GO layer, as well as the sensing performance of the last two for ethanol. It was clear that the GO layer enhanced both the sensitivity and accuracy of the FO-SPR sensor thanks to its excellent electrochemical and physical properties.

**Figure 20:**
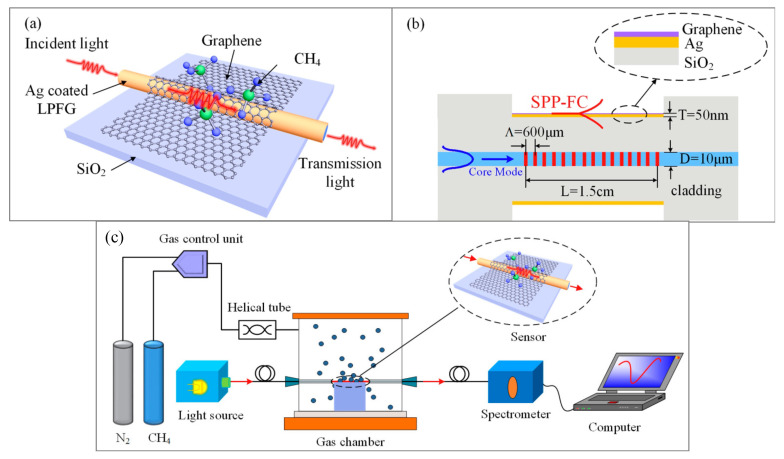
(**a**) Schematic representation of the graphene-based long period fiber grating SPR sensor. (**b**) Longitudinal section of the sensor. (**c**) The experimental setup used [223].

Wei et al. [223] proposed a long period fiber grating (LPFG) SPR sensor combined with a monolayer of graphene as sensing material. To fabricate the sensor, a single mode fiber with a core diameter of 10 µm, a cladding diameter of 125 µm, and a numerical aperture of 0.22 was used. The long period grating was first inscribed on the fiber core by a CO_2_ laser and then the SiO_2_ surface of the fiber was coated with an Ag film (50 nm thick) on top of which a monolayer of graphene was deposited by CVD. A schematic representation of the sensor structure is given in Figure 20a,b. To test the performance of the sensor chip, an experimental setup (Figure 20c) comprising a gas flow control system, a wide spectral range light source and a spectrometer was used. The LPFG SPR sensor was exposed to different concentrations of methane carried by a nitrogen gas flow. Wavelength interrogation was employed to monitor changes in the refractive index and thus detect variations in the concentration of VOCs in contact with the sensor. The obtained graphene coated LPFG SPR sensor exhibited a dose dependent linear response to methane and improved sensitivity compared to an uncoated LPFG sensor and an Ag-coated LPFG SPR sensor. The sensor also demonstrated good response repeatability and a baseline recovery (with a recovery time of 65 s). Finally, using finite element simulation, the team showed that the graphene layer enhanced the intensity of the electric field surrounding the sensing layer, which could explain the sensitivity enhancement observed in the presence of this layer.

The Table 3 summarizes the conditions for gas or VOCs detection of fiber optic SPR-based artificial olfaction systels and their performances in liquid and gas phase.

### 3.4. Grating Coupler-Based SPR Sensors

**Figure 21:**
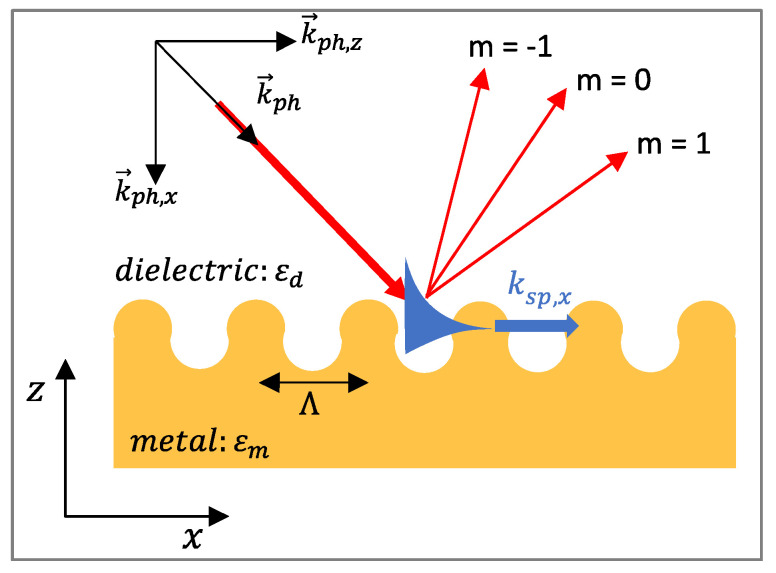
Excitation of SPs by grating coupler.

Based on light diffraction effects, the grating coupler is another approach to excite surface plasmons. This method was first observed and described by Wood [122] in 1902. Basically, when a light wave reaches a periodically distorted metal-dielectric interface, it is diffracted into a series of beams that propagate away from the surface at different angles [147] (Figure 21). Coupling occurs when the momentum component along the interface of a scattered order is equal to the propagation constant of the SPPs. The coupling condition can by expressed as [121]:

(6) $$\frac{2\pi }{\lambda }\;\sqrt{\varepsilon_{d}}\;\sin\theta +m\frac{2\pi }{\Lambda }=\;\pm Re\left\{k_{spp}\right\}$$

where ***λ*** is the wavelength of the incident p-polarized light, θ the incidence angle, *m* the diffraction order and Λ the diffraction grating period. To perform measurements using this type of SPR sensors, angular, spectral, phase or intensity interrogation can be employed.

This category of sensors is much less popular and poorly developed compared to those presented above, because they are generally less sensitive than smooth metal-film coupling based sensors (i.e., prism and optical fiber). Several theoretical and experimental studies have been carried out in attempts to improve the performance of these sensors [132,229,230]. For instance, Nazem et al. [229] recently demonstrated (theoretically and experimentally) the feasibility of a sensitive SPR sensor based on Ag-MgF_2_ grating. Similarly, Dai et al. [230] experimentally demonstrated a high sensitivity of an SPR sensor with silver rectangular grating coupling. A higher sensitivity than that of a prism-coupled SPR sensor was obtained in the negative order diffraction excitation mode. Borile et al. [231] reported a grating-coupled SPR senor integrated into a microfluidic chamber for label-free monitoring of cell adhesion and cell-surface interaction. Cai et al. [232] worked on the improvement of the sensitivity of grating-based SPR sensors by designing sharp dips of the higher diffraction orders and developing double-dips method. Finally, in the field of VOC sensing, Sambles’s group [233,234] presented a prototype gas sensor employing SPR on gratings in the beginning of 1990s. Since then, this field has not been developed much further.

## 4. Conclusions and Outlook

The reliable analysis of VOCs is of great interest in various fields. To complement traditional analytical methods (GC-MS) and biological noses, great progress has been made in the development of artificial odor detection systems such as gas sensors, olfactory biosensors, and eNs based on diverse sensing technologies. As demonstrated in this paper, propagating SPR with different coupling configurations (prism coupler, wave guide, and grating) is very efficient for such applications. In particular, prism coupler-based gas sensors have been widely studied for sensing VOCs, either in the liquid or gas phase. For VOC analysis in the liquid phase, as highlighted in this review, signal amplification strategies are necessary by selecting appropriate sensitive materials and immobilization techniques to generate reliable SPR signals. In contrast, in the gas phase, the binding of small VOCs on the sensing materials can generate reliable SPR signals with good signal/noise ratios, since the detection noise remains relatively low under such conditions. Moreover, based on SPR imaging mode, a novel generation of eNs with large-scale multiplexed arrays has been developed. Combined with peptides as sensing materials, such eNs offer exceptional performance in terms of sensitivity and selectivity, with the ability to discriminate among chiral forms of VOCs. Regarding wave guide coupler-based gas sensors, most systems use optical fiber in different configurations. They are very interesting thanks to their remote and multiplexed sensing capability, as well as their miniaturized structures. Finally, grating coupler-based gas sensors are much less popular because their sensitivity is still limited.

Although the different systems that we have presented are efficient and sensitive for the detection of VOCs, the current trend is toward the development of more miniaturized sensors. Accordingly, nano plasmonic sensors based on localized SPR are attracting more and more attention, and are being developed for different nanoscale applications including the detection of VOCs. Moreover, the sensing performance of these systems can be optimized by simply varying the size and shape of the nanostructures, which is very advantageous for sensor development. The improvement in nanofabrication processes has made it possible to explore diverse nanostructured geometries to achieve optimal LSPR nanosensors [137].

To further improve the performance (sensitivity, selectivity, and stability) of SPR-based gas sensors, olfactory biosensors, and eNs, it is essential to design novel sensing materials that are able to mimic the binding properties of biomolecules such as ORs and OBPs, but with higher stability. One trend is to use peptides as alternatives. Indeed, peptides are much more robust than proteins, cheaper to synthesize, and could potentially be integrated into industrial devices. On top of that, their selectivity towards target VOCs can be easily tuned through rational designs based on molecular modeling, virtual screening, and phage display. Finally, eNs will benefit greatly from the accelerating growth of artificial intelligence that will allow for more efficient data processing. There is no doubt that novel SPR-based gas sensors and eNs will play a more important role in the field of VOC detection and will find applications in various new domains.

## Figures and Tables

**Table 1:** SPR-based olfactory biosensors in the Kretschmann configuration for the detection of VOCs in liquid phase.

Interrogation	Amplification Strategy	Sensing Material	Performance	Refs.
Resonance angle	Desorption of the G_αolf_ subunit and possible conformation change	Rat ORI7 Human OR17-40 (Carried by nanosomes)	Conservation of the binding affinity => high selectivity Repeatability: up to eight activation cycles	[164]
Resonance angle	Desorption of the G_αolf_ subunit and conformational change	Human OR17-40 (Carried by nanosomes)	Conservation of the binding affinity => high selectivity to helional Stability: two days	[166]
Reflected light intensity	G-protein transduction cascade	Rat ORI7 (Carried by artificial olfactory cell)	Conservation of the binding affinity => high selectivity to octanal Octanal detection limit: 0.1 mM	[167]
Reflected light intensity	Possible conformational change	Three rat OBP-3 mutants	Very low detection limit in concentration: 200 pM for β-ionone and in molecular weight of VOCs: 100 g/mol for hexanal Higher selectivity at low concentration of VOCs Repeatability from measurement to measurement and from chip to chip Lifespan up to almost two months	[177]
Reflected light intensity	Possible conformational change	Toluene binding domain (TBD)	High selectivity and sensitivity to toluene (detection limit: 15.62 µM)	[178]

**Table 2:** SPR-based artificial olfaction systems in the Kretschmann configuration for the detection of VOCs in gas phase.

Artificial Olfaction System	Interrogation	Sensing Material	Performance	Refs.
Electronic nose	Reflected light intensity (Imaging)	Small peptides	octanol detection limit (DL): below 1 ppm High discrimination ability (one carbon atom resolution) Stability: at least three months Good repeatability	[185]
Electronic nose	Reflected light intensity (Imaging)	Penta-peptides and hairpin DNA	High discrimination ability (one carbon atom resolution)	[191]
Gas sensor	Resonance wavelength	Cavitands	High selectivity and sensitivity to DMMP (DL: 16 ppb)	[192]
Gas sensor	Reflected light intensity	Three nitro-substituted heterocalix[4]arenes thin films	High selectivity and sensitivity to acetone (DL: 3.8 ppm) Fast and reversible response (few seconds) Repeatability: from chip to chip and up to four injection cycles	[194]
Gas sensor	Reflected light intensity and resonance angle	Poly(methylmethacrylate) film	High selectivity and sensitivity to benzene Fast and reversible response	[195]
Gas sensor	Resonance angle	Acrylic acid and styrene thin film	Acrylic acid film: good selectivity to ammonia (DL: several ppm) and amines (trimethylamine and trimethylamine Styrene film: poor selectivity to tested gases	[198]
Gas sensor	Resonance angle	Films of polythiophene (PT) or gold nanoparticles capped with conjugated oligothiophenes (NPOT)	PT film: responded to alcohol and toluene NPOT film: responded only to alcohols => high selectivity	[200]
Gas sensor	Resonance angle	Reduced graphene oxide/maghemite nanocomposite film	High selectivity and sensitivity to acetone	[201]

**Table 3:** Fiber optic SPR-based artificial olfaction systems for the detection of VOCs in liquid and gas phase.

Artificial Olfaction System	Fiber Type	Sensing Material	Performance	Sensing Medium	Refs.
Olfactory biosensor	Plastic fiber	Pig odorant binding protein	High selectivity and to butanal sensitivity (detection limit (DL): 25 µM)	Liquid	[215]
Gas sensor	Glass fiber	Zeolitic imidazolate framework (ZIF-8 and ZIF-93)	ZIF-8: high sensitivity to methanol (DL: 2.5 ppm)	Gas	[217]
Gas sensor	Plastic clad silica fiber	Graphene-carbon nanotubes/poly(methyl methacrylate) (GCNT/PMMA) hybrid composites, reduced graphene oxide, carbon nanotubes, reduced graphene oxide-carbon nanotubes	GCNT/PMMA exhibited the highest sensitivity and selectivity to methane compared to the other sensing materials tested DL: 10 ppm	Gas	[220]
Gas sensor	Photonic crystal fiber	Pd-WO_3_ film and a kind of ultraviolet curable fluoro-siloxane nanofilm with the inclusion of cryptophane A	The concentration of methane and hydrogen in a gas mixture could be accurately measured using polarization filtering	Gas	[221]
Gas sensor	Fiber Bragg grating	Graphene oxide (GO)	The GO layer enhances the sensitivity to ethanol compared to bare gold	Liquid	[222]
Gas sensor	Long Period Fiber Grating (LPFG)	Graphene	In presence of graphene, the sensitivity to methane is improved 2.96 and 1.31 times with respect to the traditional LPFG sensor and Ag-coated LPFG SPR sensor, respectively Fast response (50 s) and recovery (65 s) times Good repeatability	Gas	[223]

## Data Availability

Not applicable.

## Abbreviations

ATR	attenuated total reflection
BAW	bulk acoustic wave
BTEX	benzene, toluene, ethylbenzene and m-xylene
CCP	composite conducting polymer
CNT	reduced graphene oxide-carbon nanotube
CP	conducting polymer
CVD	chemical vapor deposition
DL	detection limit
DMMP	dimethylmethylphosphonate
EIS	electrochemical impedance spectroscopy
eN	electronic nose
FBG	fiber Bragg grating
FET	field effect transistor
FO-SPR	fiber optic-SPR
FOS	fiber optic sensor
GC-MS	gas chromatography-mass spectrometry
GCNT	reduced graphene oxide-carbon nanotubes
GLAD	glancing-angle deposition
GO	graphene oxide
GPCR	G protein coupled receptors
GTP	guanosine-5′-triphosphate
ICP	intrinsic conducting polymer
IDT	inter-digitated transducer
LPFG	long period fiber grating
LSPR	localized SPR
MIP	molecularly imprinted polymer
MO-SPR	magneto-optical SPR
MOF	metal organic framework
MOS	metal oxide semiconductor
NP	nanoparticles
NPOT	NP/oligothiophene
OBP	odorant binding protein
OR	olfactory receptor
PCF	photonic crystal fiber
PMMA	poly(methylmethacrylate)
POF	plastic optical fiber
ppb	parts per billion
ppm	parts per million
PT	polythiophene
QCM	quartz crystal microbalance
rGO	reduced GO
SAW	surface acoustic wave
SiNW	FET silicon nanowire FET
SMF	single mode fiber
SP	surface plasmon
SPP	surface plasmon polariton
SPR	surface plasmon resonance
SPW	surface plasma wave
TBD	toluene binding domain
TECS	technology enhanced clad silica
TIR	total internal reflection
TM	transverse-magnetic
TNT	2,3,6-trinitrotoluene
VOC	volatile organic compound
ZIF	zeolitic imidazolate framework
